# Supplementary material for: Immune-regulated IDO1-dependent tryptophan metabolism is source of one-carbon units for pancreatic cancer and stellate cells
Source: Mol Cell. 2021 Jun 3;81(11):2290–2302.e7. doi: 10.1016/j.molcel.2021.03.019 (PMC8189438; doi:10.1016/j.molcel.2021.03.019)
Supplement: Document S1. Figures S1–S5 [file mmc1.pdf]

Molecular Cell, Volume *81*

Supplemental information

Immune-regulated IDO1-dependent tryptophan metabolism is source of one-carbon units for pancreatic cancer and stellate cells

Alice Clare Newman, Mattia Falcone, Alejandro Huerta Uribe, Tong Zhang, Dimitris Athineos, Matthias Pietzke, Alexei Vazquez, Karen Blyth, and Oliver David Kenneth Maddocks

Supplemental Figure 1.

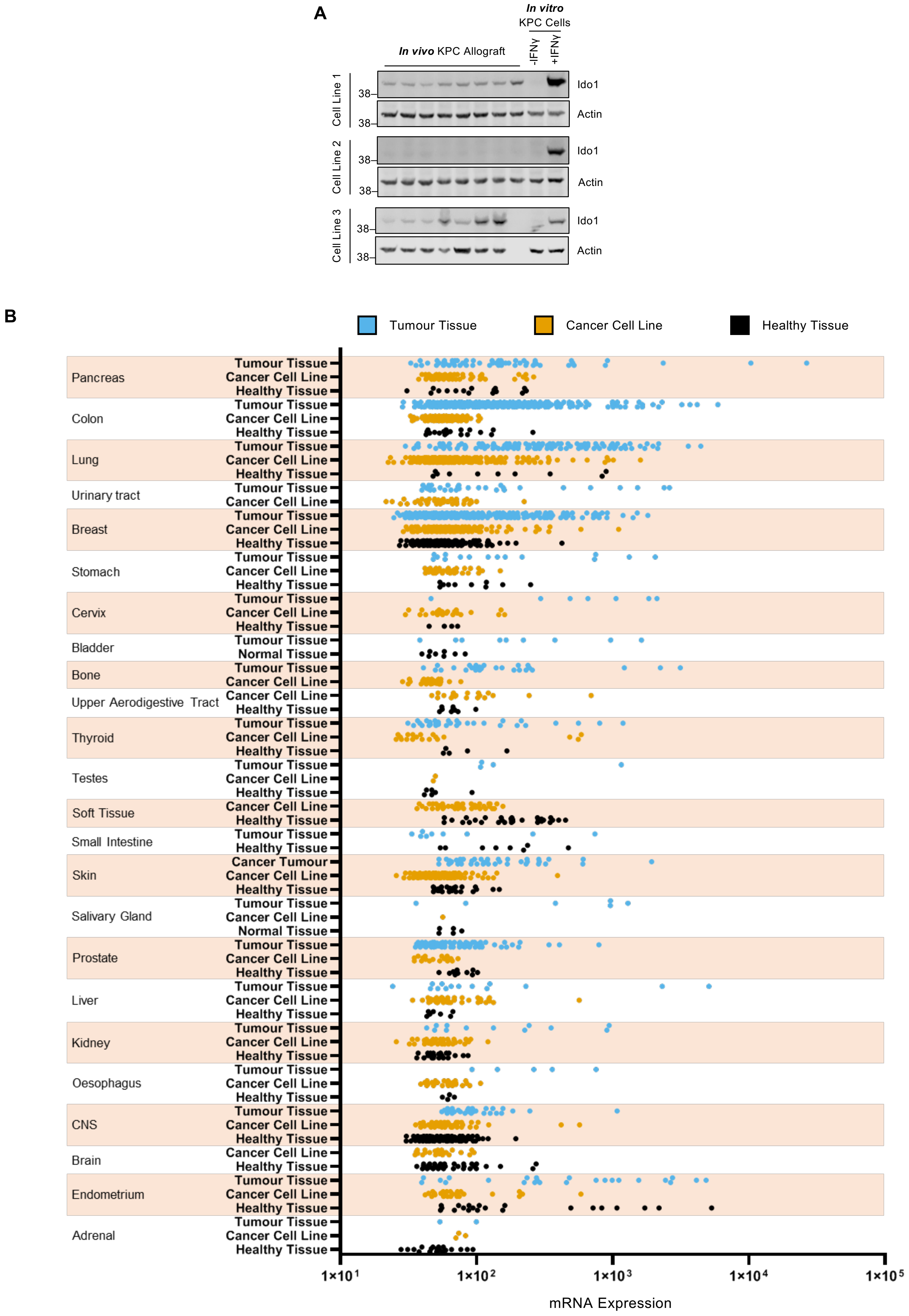

Supplemental Figure 1.

IDO1 expression in mouse and human tumours. Related to Figure 1.

A, KPC cells were isolated from C57Bl6/J *Pdx1*-cre;*Kras*$^{G12D/+}$;*Trp53*$^{R172H/+}$ mice and either treated in culture with mouse IFNγ (1ng/ml) for 24h or subcutaneously injected into the flank of C57Bl6/J mice to form tumours. Cell and tumour lysates were subjected to western blot for the indicated proteins. **B**, Data extracted from the MERAV database showing the relative abundance of IDO1 mRNA from microarray data across a range of human cell lines and tumour tissue (http://merav.wi.mit.edu/; Shaul, Y.D., Yuan, B., Thiru, P., Nutter-Upham, A., McCallum, S., Lanzkron, C., Bell, G.W., and Sabatini, D.M. (2016). MERAV: a tool for comparing gene expression across human tissues and cell types. Nucleic acids research *44*, D560-566).

Supplemental Figure 2.

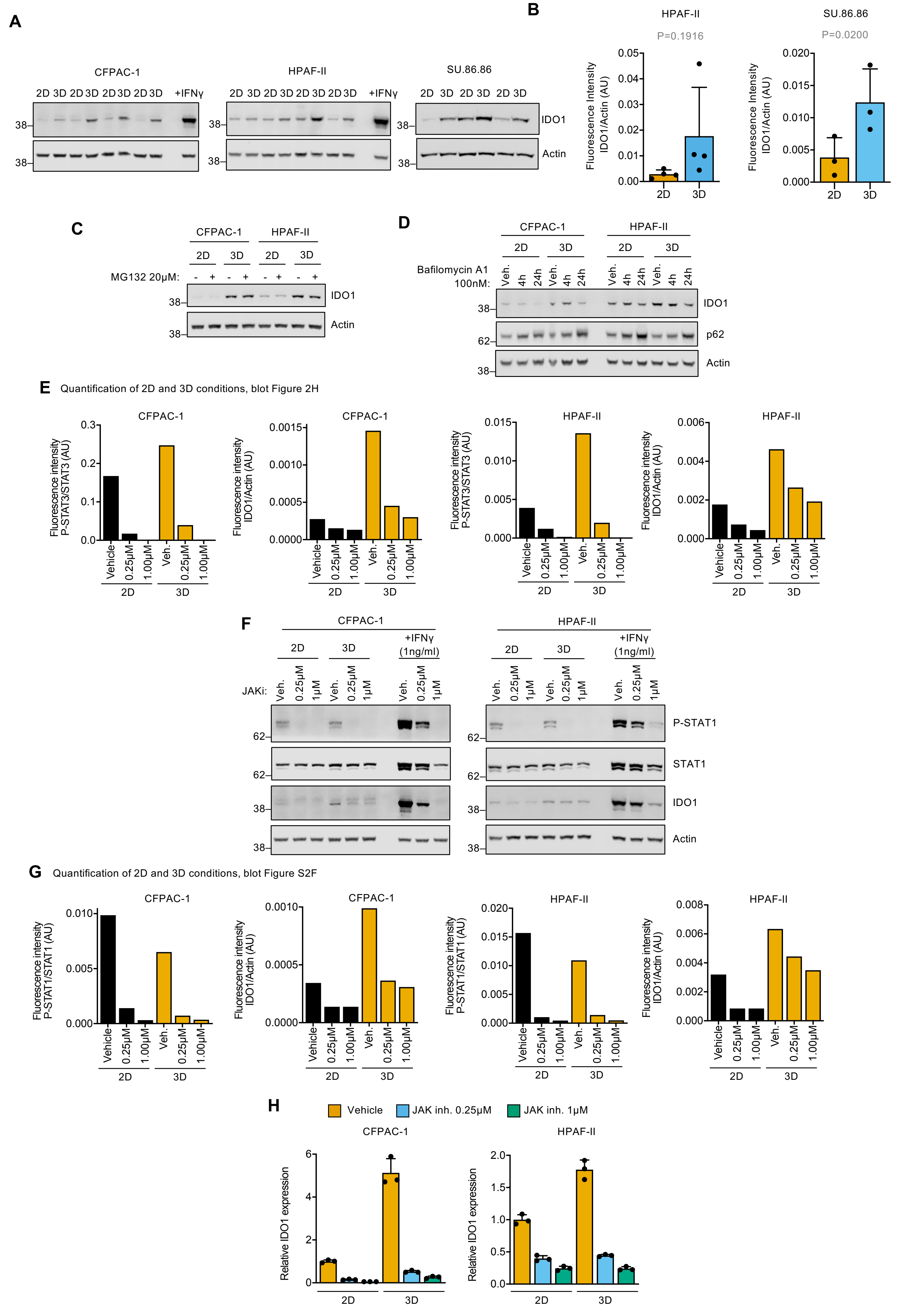

Supplemental Figure 2.

Regulation of IDO1 expression by attachment independent 3D growth. Related to Figure 2.

A, B, CFPAC-1, HPAF-II and SU.86.86 cells were either growth in normal tissue culture plates (2D) or in ultra-low-attachment tissue culture plates (3D) for 24h, or cultured in 2D and treated with 1ng/ml IFNγ. Lysates were (**A**) analysed by western blot for the indicated proteins and (**B**) band intensity of IDO1 relative to actin (loading control) quantified using LiCor infra-red scanner (n=3 or 4 independent experiments, paired 2-sided t-test, P-value shown, bars = SD). **C-E**, CFPAC-1 and HPAF-II cells were grown in 2D or 3D conditions for 24 hours and lysates analysed by western blot for indicated proteins (**C**) after 16h treatment with proteasome inhibitor MG132 (20μM) or vehicle-only control (**D**) after treatment for the indicated times with lysosome inhibitor bafilomycin A1 (100nM) or vehicle-only control (p62 used as positive control for lysosomal regulation). **E,** Quantification of IDO1 and phospho-STAT3 band intensities using LiCor infra-red laser scanner for blot shown in Figure 2H. **F,** CFPAC-1 and HPAF-II cells were grown in 2D or 3D conditions for 24 hours and lysates analysed by western blot for indicated proteins after 16h treatment with JAKi (at indicated concentrations), vehicle-only control or IFNγ (1ng/ml). **G**, Quantification of IDO1 and phospho-STAT1 band intensities using LiCor infra-red laser scanner for blot shown in Figure S2F. **H**, mRNA was extracted from CFPAC-1 and HPAF-II cells and subjected to qRT-PCR (n=3 biological replicates, bars = SD).

Supplemental Figure 3.

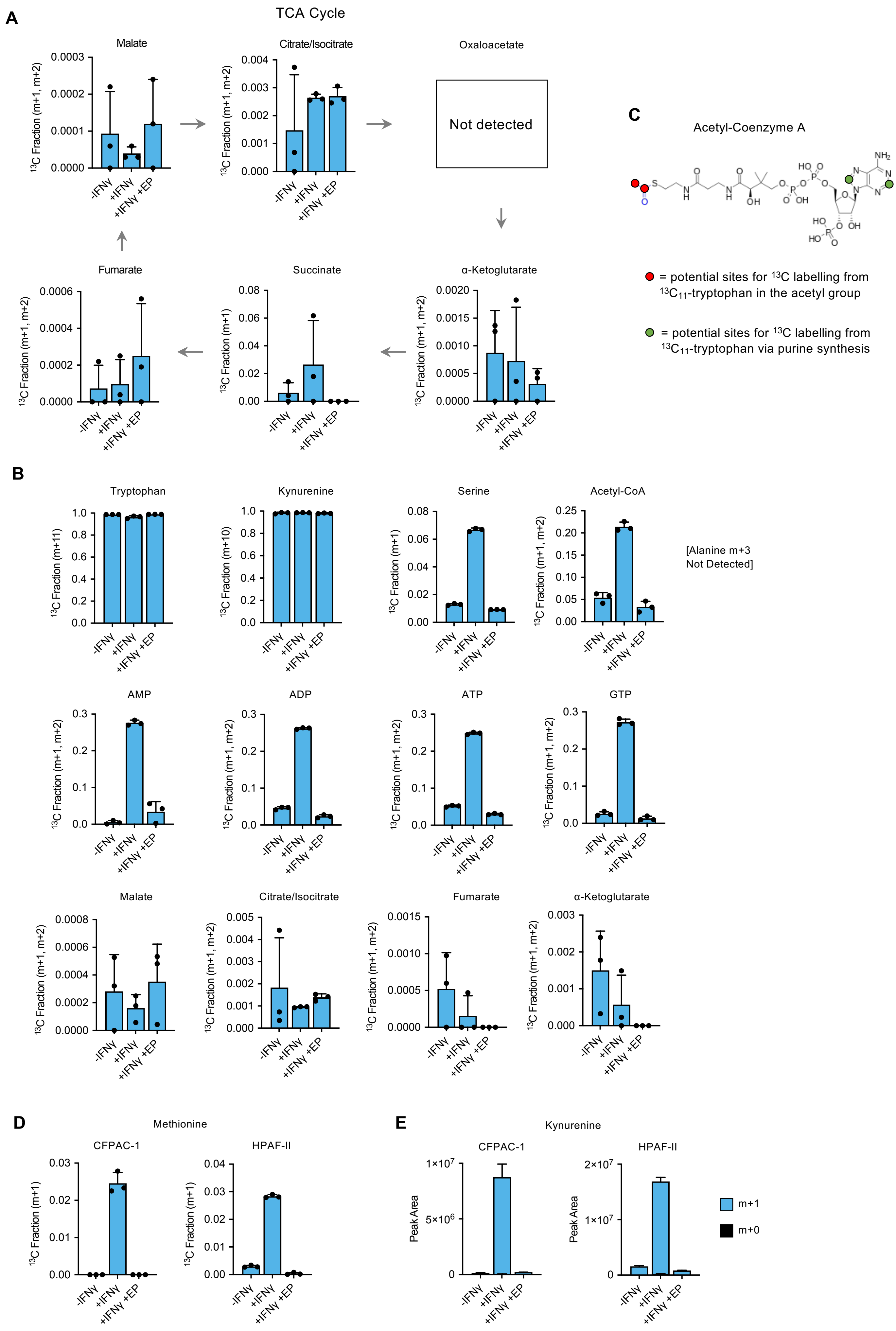

Supplemental Figure 3.

IDO1-dependent tryptophan metabolism analysed by LCMS. Related to Figure 3.

A & B, CFPAC-1 (**A**) and HPAF-II (**B**) cells were grown with or without human IFNγ (1ng/ml), or with IFNγ and IDO1 inhibitor Epacadostat for 24h in in the presence of $^{13}C_{11}$-tryptophan. Intracellular metabolites were analysed by LCMS and corrected for natural ^{13}C abundance (n=3 biological replicates, bars = SD). **C**, The potential positions of tryptophan derived carbons are shown in a structural diagram of Acetyl-CoA. **D,** methionine levels detected by LCMS in the same experiments described in A & B above. **E**, Raw peak area values for kynurenine detected by LCMS are shown for the same experiments described in A & B above.

Supplemental Figure 4.

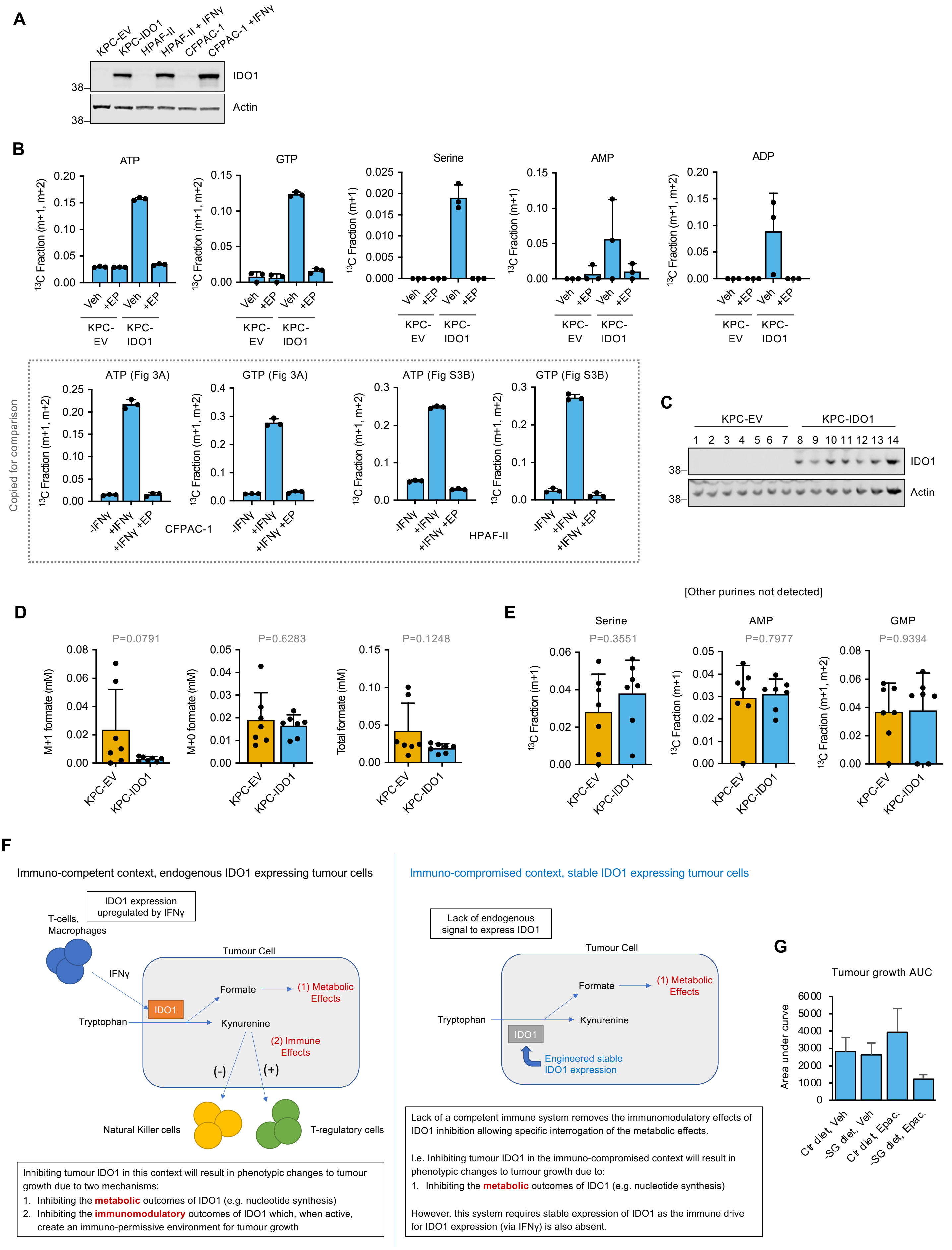

Supplemental Figure 4.

***In vivo* tryptophan metabolism. Related to Figure 4.**

A, KPC cell line 2 (Figure 1E & S1A) from C57Bl6/J Pdx1-cre;*Kras*$^{G12D/+}$;*Trp53*$^{R172H/+}$ mice were engineered to stably express IDO1 (KPC-IDO1) or empty-vector control (KPC-EV), protein lysates from cells were analysed by western blot for IDO1 expression alongside CFPAC-1 and HPAF-II cells which had been treated with human IFNγ (1ng/ml) for 24h. **B**, KPC-EV and KPC-IDO1 cells were grown with or without IDO1 inhibitor Epacadostat for 24h in in the presence of $^{13}C_{11}$-tryptophan. Intracellular metabolites were analysed by LCMS and corrected for natural ^{13}C abundance (n=3 biological replicates, bars = SD). ATP and GTP LCMS data for IFNγ/epacadostat treated CFPAC-1 and HPAF-II cells (copied from Figures 3A and S3B) is shown for comparison. **C-E**, KPC-EV and KPC-IDO1 cells were injected subcutaneously into the flanks of C57Bl6/J mice (as shown and used in Figure 4A &B), once tumours had formed the mice were given a single by intraperitoneal injection of 800µl 120mM $^{13}C_{11}$ tryptophan and harvested after 3h. Tumour tissue was excised and analysed by immunoblotting for the indicated proteins (**C**). Serum was analysed by GCMS for formate (**D**) and LCMS for serine and purine nucleotides (**E**) (n=7 mice in each group, bars are SD). **F,** Cartoon illustrating the rationale for use of immunocompromised mice with stable IDO1-expressing (KPC-IDO1) tumour cells to specifically interrogate the metabolic effects of IDO1 inhibition on tumour growth. **G,** Area under the curve analysis was performed on the tumour volume data presented in Figure 4E & F, bars are standard error of mean.

Supplemental Figure 5.

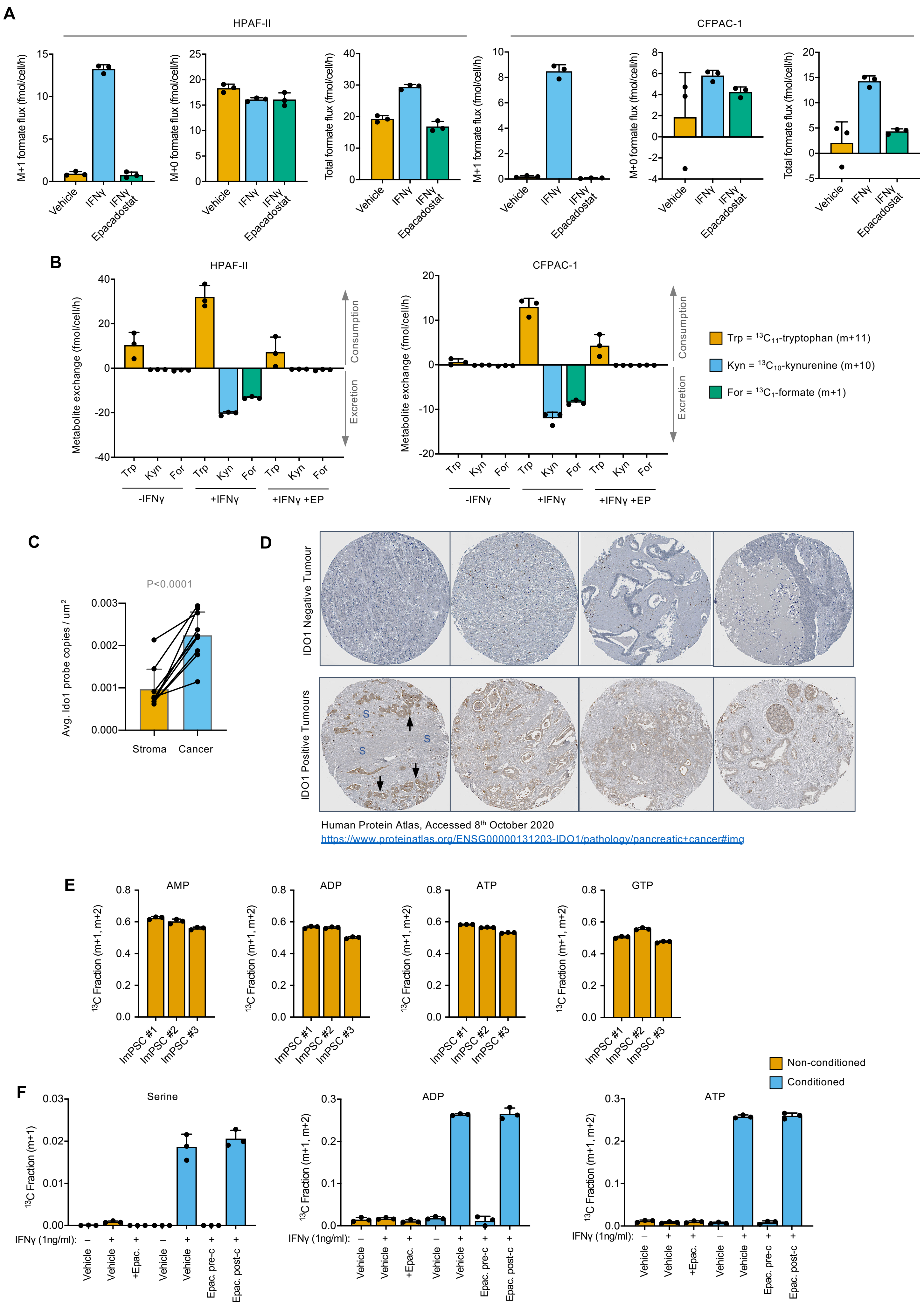

Supplemental Figure 5.

Formate metabolism in PDAC and pancreatic stellate cells. Related to Figure 5.

A & B, HPAF-II and CFPAC-1 cells were treated with IFNγ (1ng/ml) with or without epacadostat (1µM) and/or vehicle only controls in the presence of $^{13}C_{11}$-tryptophan for 24h. Cell were grown in complete medium containing serine and glycine (both 0.4mM) and $^{13}C_{11}$-tryptophan 0.4mM. Media samples were analysed by a targeted GCMS for extracellular formate (A) and by LCMS for tryptophan and kynurenine (B). (n=3 biological replicates, bars = SD). GCMS data for unlabelled (m+0), labelled (m+1) and total (m+0 + m+1) formate is shown in (A). LCMS data for labelled tryptophan (m+11) and kynurenine (m+10) are shown in (B), alongside labelled formate (m+1) data reproduced from (A) for comparison. **C,** Formalin fixed paraffin embedded tissue sections from tumours shown in Figure 1B were analysed by RNA-Scope for IDO1 transcript expression (n=10 tumours, paired 2-sided T-test, P-value shown). Contiguous tissue sections were stained by immunohistochemistry for a cancer cell marker (EpCAM) to differentiate stromal cells from cancer cells. **D,** Images of pancreatic cancer tumours stained by immunohistochemistry for IDO1 were downloaded from the Human Protein Atlas. 'S' denotes stroma, arrow heads indicate PDAC cells embedded within the stroma **E**, Three separate immortalised murine pancreatic stellate cell lines (ImPSC#1, ImPSC#2 & ImPSC#3) were cultured for 24h in the presence of $^{13}C_1$-formate and intracellular metabolites were analysed by LCMS (n=3 biological replicates, bars = SD). **F**, CFPAC-1 cells were treated with IFNγ (1ng/ml) and/or epacadostat (1µM) and/or vehicle only in the presence of $^{13}C_{11}$-tryptophan. Conditioned media were collected after 24h and ImPSC#2 cells were cultured in this media, or in non-conditioned treatment-matched media. After 24h, intracellular metabolites were analysed by LCMS (n=3 biological replicates, bars = SD).
