# Supplementary material for: Immune-regulated IDO1-dependent tryptophan metabolism is source of one-carbon units for pancreatic cancer and stellate cells
Source: Mol Cell. 2021 Jun 3;81(11):2290–2302.e7. doi: 10.1016/j.molcel.2021.03.019 (PMC8189438; doi:10.1016/j.molcel.2021.03.019)
Supplement: Document S2. Article plus supplemental information [file mmc2.pdf]

Molecular Cell

Article

Immune-regulated IDO1-dependent tryptophan metabolism is source of one-carbon units for pancreatic cancer and stellate cells

Graphical abstract

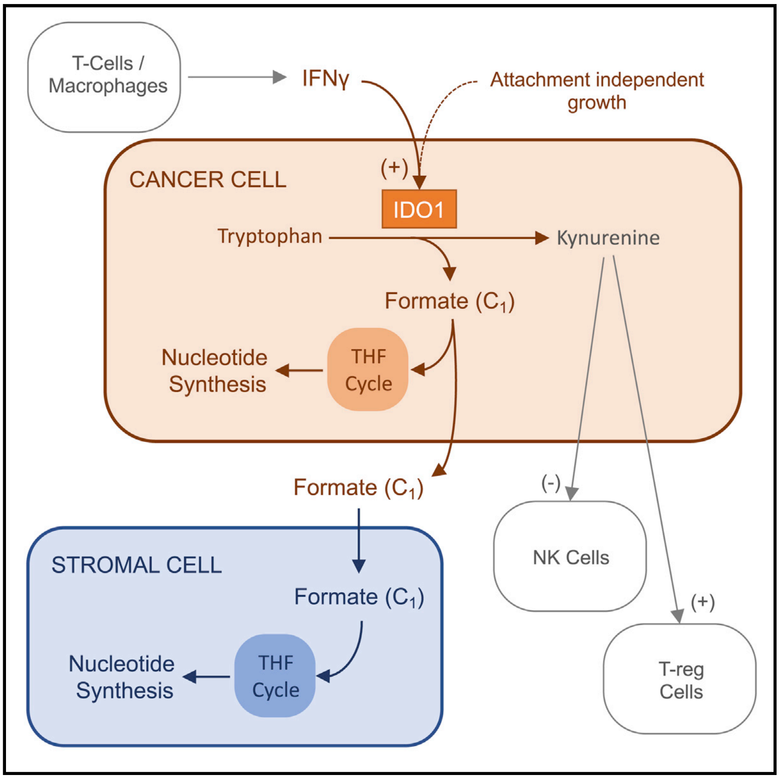

Highlights

- IFNγ and attachment-independent growth promote IDO1 expression in PDAC cells
- Metabolism of tryptophan by IDO1 contributes one-carbons to the THF cycle
- Tryptophan can substitute for serine as a one-carbon source
- Serine and glycine restriction enhances the anti-tumor activity of an IDO1 inhibitor

Authors

Alice Clare Newman, Mattia Falcone, Alejandro Huerta Uribe, ..., Alexei Vazquez, Karen Blyth, Oliver David Kenneth Maddocks

Correspondence

oliver.maddocks@glasgow.ac.uk

In brief

Newman et al. studied the metabolic outcomes of immune-regulated IDO1 expression in pancreatic cancer and stellate cells, reporting that IDO1-dependent tryptophan metabolism is a bona fide one-carbon source for folate-dependent nucleotide synthesis. Tryptophan substituted for serine as a one-carbon donor, with serine restriction improving the anti-tumor activity of IDO1 inhibitor epacadostat.

Newman et al., 2021, Molecular Cell *81*, 2290–2302
June 3, 2021 © 2021 The Authors. Published by Elsevier Inc.
https://doi.org/10.1016/j.molcel.2021.03.019

CellPress
OPEN ACCESS

Molecular Cell

Article

Immune-regulated IDO1-dependent tryptophan metabolism is source of one-carbon units for pancreatic cancer and stellate cells

Alice Clare Newman,[1] Mattia Falcone,[1,3] Alejandro Huerta Uribe,[1,3] Tong Zhang,[1,4] Dimitris Athineos,[2] Matthias Pietzke,[2,5] Alexei Vazquez,[1,2] Karen Blyth,[1,2] and Oliver David Kenneth Maddocks[1,6,*]
[1]Institute of Cancer Sciences, Wolfson Wohl Cancer Research Centre, University of Glasgow, Switchback Road, Glasgow G61 1QH, UK
[2]Cancer Research UK Beatson Institute, Switchback Road, Glasgow G61 1BD, UK
[3]These authors contributed equally
[4]Present address: Novartis Institutes for BioMedical Research, Shanghai, China
[5]Present address: Max Delbruck Centre for Molecular Biology, 13092 Berlin, Germany
[6]Lead contact
*Correspondence: oliver.maddocks@glasgow.ac.uk
https://doi.org/10.1016/j.molcel.2021.03.019

SUMMARY

Cancer cells adapt their metabolism to support elevated energetic and anabolic demands of proliferation. Folate-dependent one-carbon metabolism is a critical metabolic process underpinning cellular proliferation supplying carbons for the synthesis of nucleotides incorporated into DNA and RNA. Recent research has focused on the nutrients that supply one-carbons to the folate cycle, particularly serine. Tryptophan is a theoretical source of one-carbon units through metabolism by IDO1, an enzyme intensively investigated in the context of tumor immune evasion. Using *in vitro* and *in vivo* pancreatic cancer models, we show that IDO1 expression is highly context dependent, influenced by attachment-independent growth and the canonical activator IFNγ. In IDO1-expressing cancer cells, tryptophan is a bona fide one-carbon donor for purine nucleotide synthesis *in vitro* and *in vivo*. Furthermore, we show that cancer cells release tryptophan-derived formate, which can be used by pancreatic stellate cells to support purine nucleotide synthesis.

INTRODUCTION

Cancer cells adapt their metabolism to support proliferation and survival (Hanahan and Weinberg, 2011). A better understanding of cancer-specific metabolic changes is key for improved cancer treatment. One-carbon metabolism has been a target of cancer therapy since the 1940s (Newman and Maddocks, 2017a, 2017b) and encompasses a collection of metabolic pathways that enable cells to generate and use molecules containing single carbons. One-carbon units are carried and activated for use by tetrahydrofolates (THFs), derived from dietary folate. Cells require one-carbon units to support nucleotide synthesis, methylation reactions, and reductive metabolism.

The non-essential amino acid serine is considered the predominant source of one-carbon units (Ducker et al., 2016; Labuschagne et al., 2014). Serine is obtained either by *de novo* synthesis via the serine synthesis pathway (SSP) or by uptake from the extracellular environment. Some cancer cells display increased SSP enzyme expression (Locasale et al., 2011; Possemato et al., 2011; Sullivan et al., 2019b), whereas others rely predominantly on serine uptake. Strategies to limit serine availability, either by the inhibition of serine synthesis (Pacold et al., 2016; Possemato et al., 2011) or the dietary restriction of serine (Baksh et al., 2020; LeBoeuf et al., 2020; Maddocks et al., 2013, 2017; Muthusamy et al., 2020), have shown promise as an anti-cancer therapy using preclinical models. Serine hydroxymethyltransferases (SHMT1 and SHMT2) directly catalyze the conversion of serine into glycine and the release of a one-carbon, which enters the THF cycle. Targeted strategies to inhibit SHMT enzymes and the downstream THF cycle enzymes controlling the utilization of serine-derived carbons have had limited success in inhibiting cancer cell proliferation, chiefly due to metabolic plasticity (Ducker et al., 2016).

Besides serine, the amino acids glycine, histidine, and tryptophan are potential one-carbon donors. Glycine can theoretically provide one-carbon units through the glycine cleavage system (GCS) (Jain et al., 2012), although the relevance of this in cancer cells is unclear (Kim et al., 2015; Labuschagne et al., 2014). Histidine catabolism can also yield one-carbon units and can sensitize cancer cells to anti-folate treatment by decreasing free THF pools (Kanarek et al., 2018). Improved understanding of how cancer cells obtain and use one-carbon units can therefore offer new opportunities to improve anti-cancer therapy.

As an essential amino acid, tryptophan is critical for protein synthesis and is a precursor for 5-hydroxytryptamine and kynurenine production. In the kynurenine pathway, the initial and rate-limiting step is the conversion of tryptophan to formyl-kynurenine. Three enzymes are capable of catalyzing this

 © 2021 The Authors. Published by Elsevier Inc.
This is an open access article under the CC BY license (http://creativecommons.org/licenses/by/4.0/).

reaction: IDO1, IDO2, and TDO. Both IDO2 and TDO have low expression levels and limited tissue specificity (Hornyák et al., 2018). Therefore, IDO1 is considered the predominant form and has been widely studied, including its activation by the immune cytokine interferon γ (IFNγ) (Katz et al., 2008; Prendergast, 2008). Formyl-kynurenine spontaneously forms kynurenine, with the release of a molecule of formate. Formate can enter the one-carbon cycle by directly reacting with THF, and it is via this pathway that tryptophan can serve as a one-carbon donor. However, it is unknown whether this process is active in cancer cells.

IDO1-dependent tryptophan metabolism in cancer has been investigated predominantly in the context of immune regulation and immunotherapy. High IDO1 expression is associated with poor prognosis in a range of cancers (Yu et al., 2018). IDO1 activity depletes tryptophan and increases kynurenine in the tumor microenvironment, causing a range of effects on immune cells. Tryptophan depletion decreases tumor-infiltrating T cell activity, possibly due to GCN2 kinase activity (Munn et al., 2005), although this has been disputed (Sonner et al., 2016). Kynurenine itself decreases effector T cell proliferation (Terness et al., 2002) and supports the differentiation of immunosuppressive T-regulatory cells through the binding of the aryl hydrocarbon receptor (Mezrich et al., 2010). Overall, these tumor microenvironmental effects are reported to provide an immunologically permissive environment for tumor growth. Reflecting the recent success of immunotherapy agents, IDO1 inhibitors such as epacadostat (Liu et al., 2010) have entered trials (clinical trials.gov returns 60 trials with epacadostat at the time of writing). However, several trials have returned disappointing results (Garber, 2018), stimulating efforts to better understand the functions of IDO1 and improve the efficacy of these inhibitors.

Despite a rich literature addressing how IDO1-driven tryptophan metabolism affects the behavior of immune cells, knowledge of how this pathway influences metabolic pathways within cancer cells themselves is largely absent. This is surprising, given that the kynurenine pathway has several metabolic outputs with widely known importance for cancer metabolism: reactive oxygen species (superoxide), one-carbon metabolism, synthesis of $NAD(P)^+$, synthesis of alanine, and entry of carbons (via α-ketoadipate) into the tricarboxylic acid (TCA) cycle.

In the present study, we sought to investigate the metabolic consequences of IDO1-driven tryptophan metabolism in the context of pancreatic ductal adenocarcinoma (PDAC). PDAC tumors are extremely aggressive, with poor clinical outcomes. Characteristically, these tumors exhibit hypovascularization and deranged metabolism and contain a large proportion of complex stroma. Non-cancerous stromal stellate cells can support tumor cell metabolism through the provision of nutrients such as alanine (Sousa et al., 2016). Unlike other tumor models, PDAC-bearing (KPC) mice are unresponsive to serine restriction (Maddocks et al., 2017). There is evidence that this may be due to the enhanced *de novo* synthesis of serine (Maddocks et al., 2017), but an additional contributing factor may be an ability to use alternative one-carbon sources such as tryptophan. Intriguingly, it has recently been reported that tryptophan is one of the most depleted nutrients in interstitial fluid within KPC PDAC tumors (Sullivan et al., 2019a).

Analysis of public data shows that several tumor types, including pancreatic cancer, have high-IDO1-expressing subsets, and we show that IDO1 is expressed in genetically engineered mouse models for PDAC. We find that IDO1 expression is not well represented in standard *in vitro* cell culture conditions, but can be induced by the canonical activator IFNγ or by culture in low attachment conditions. We show that when IDO1 is expressed by cancer cells, it promotes the generation of one-carbon units from tryptophan that are used in *de novo* purine nucleotide synthesis. Under low serine conditions, tryptophan can act as an alternative one-carbon source to support proliferation, and that its combination with dietary serine and glycine restriction can increase the anti-tumor efficacy of the IDO1 inhibitor epacadostat. We also show that tryptophan-derived formate can be released by cancer cells. Intriguingly, we find that pancreatic stellate cells (a key component of the tumor stroma) can capture this exogenously derived formate and, in similarity to cancer cells, channel it into *de novo* nucleotide synthesis.

RESULTS

PDAC cells express IDO1 in a context-dependent manner

To perform metabolic analyses on cells expressing physiologically relevant levels of IDO1, we sought to evaluate the expression of IDO1/Ido1 in pancreatic cancer cells across a range of *in vitro* and *in vivo* contexts (Figure 1A). Direct analysis of pancreatic tumor tissue from *Pdx1*-Cre;LSL-*Kras*$^{G12D/+}$;*Trp53*$^{fl/+}$ and *Pdx1*-Cre;LSL-*Kras*$^{G12D/+}$;LSL-*Trp53*$^{R172H/+}$ mice showed that tumors had increased Ido1 expression versus normal pancreas tissue, and that certain tumors expressed high levels of Ido1 (Figures 1B and 1C). Compared to genetically engineered mouse model (GEMM) tumor tissue, tumor-derived primary KPC cells cultured under normal *in vitro* conditions displayed undetectable Ido1 (Figure 1D). Addition of the murine form of the immune cytokine IFNγ, a canonical activator of Ido1, increased Ido1 expression *in vitro*. As expected, the human form of IFNγ did not affect Ido1 expression in murine cells (Figure 1D).

To assess whether *in vivo* growth could restore Ido1 expression, we injected mixed background or pure C57Bl6/J KPC cells into mice as subcutaneous allografts in three separate experiments. In the first experiment, mixed background primary KPC cells showed very low Ido1 expression when injected into immunocompromised CD1-nude mice (Figure 1D). In contrast, in the second experiment, the growth of 3 C57Bl6/J KPC cell lines in immunocompetent syngeneic mice showed several instances of strong Ido1 expression for 2 of 3 cell lines (Figure S1A), albeit with intra-animal variability, as seen with autochthonous KPC models in Figure 1B. For the third experiment, we implanted the 3 C57Bl6/J KPC cell lines in immunocompetent (C57Bl6/J) and immunocompromised (CD-1 nude) mice simultaneously and directly compared Ido1 expression (using cell line no. 2 *in vitro* as a western blot control for Ido1 expression) (Figure 1E). This side-by-side experiment clearly showed a lack of Ido1 expression in immunocompromised mice, but several instances of robust Ido1 expression in immunocompetent mice, with Ido1 levels *in vivo* comparable to *in vivo* growth +IFNγ. Overall, these data suggest that under normal (immunocompetent), conditions

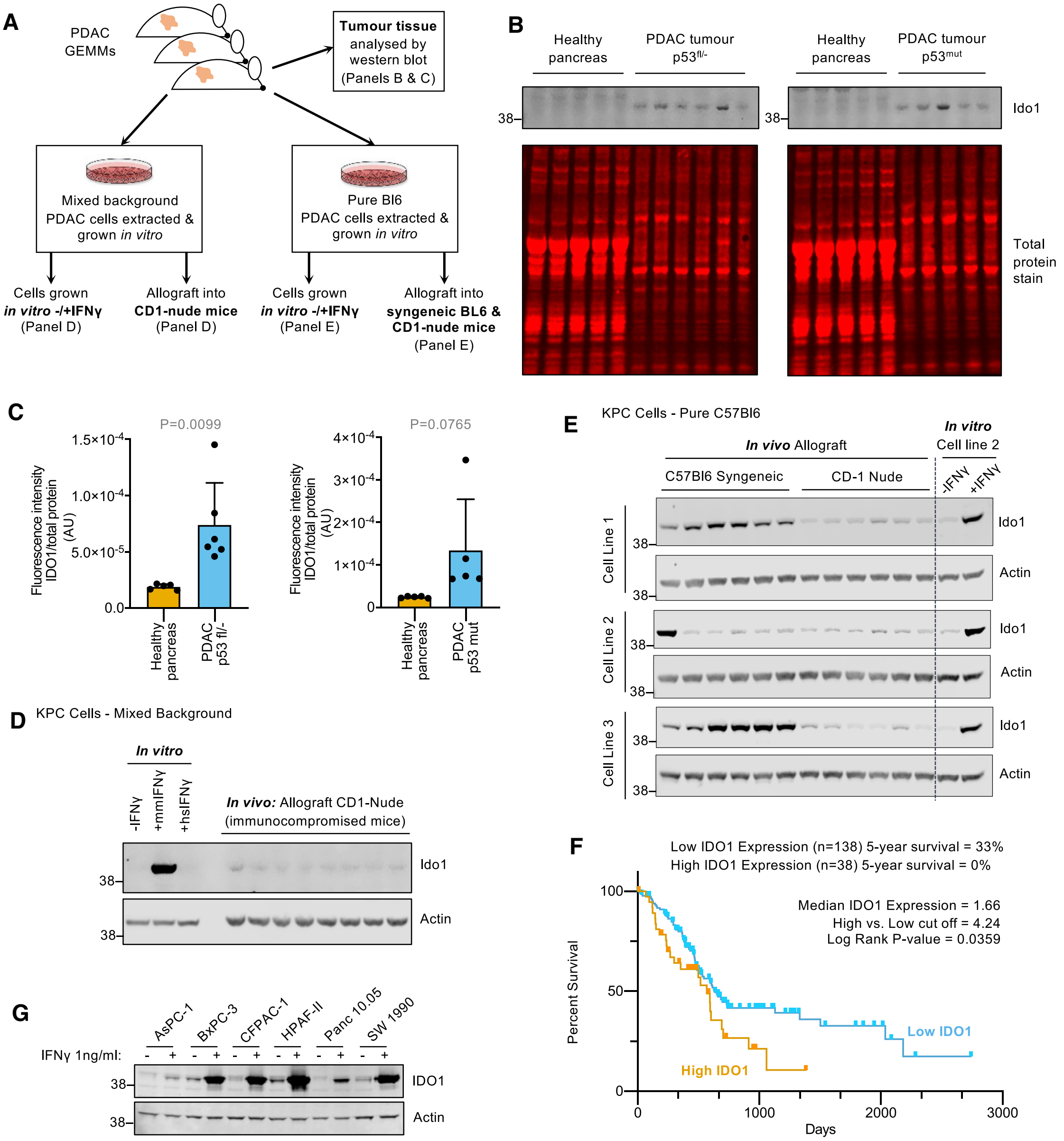

Figure 1. Tumor expression of Ido1 in KPC PDAC models requires an intact immune system *in vivo*

(A) Schematic diagram detailing the methods used to analyze Ido1 expression in mouse models of PDAC.

(B and C) Tumors from *Pdx1*-Cre;*Kras*$^{G12D/+}$;*Trp53*$^{fl/+}$ and *Pdx1*-Cre;*Kras*$^{G12D/+}$;*Trp53*$^{R172H/+}$ mice and healthy pancreas tissue from non-Cre-expressing isogenic control mice analyzed by western blots (B) quantified using a Li-Cor infrared scanner (C) (healthy pancreas n = 5, *Pdx1*-Cre;*Kras*$^{G12D/+}$;*Trp53*$^{fl/+}$ tumors n = 6, *Pdx1*-Cre;*Kras*$^{G12D/+}$;*Trp53*$^{R172H/+}$ tumors n = 5, p values calculated with unpaired 2-sided t test, bars are SDs).

(D) KPC cell line isolated from a mixed-background *Pdx1*-Cre;*Kras*$^{G12D/+}$;*Trp53*$^{R172H/+}$ tumor was either grown *in vitro* culture (with or without human or mouse IFNγ (1 ng/mL) for 24 h), or subcutaneously injected into the flank of CD1-nude mice to form tumors. Cell and tumor lysates were analyzed for the indicated proteins.

(E) Three KPC cell lines isolated from C57Bl6/J *Pdx1*-Cre;*Kras*$^{G12D/+}$;*Trp53*$^{R172H/+}$ mice were injected into the flanks of C57Bl6/J mice or CD-1-nude mice to form tumors. Cell and tumor lysates were analyzed for the indicated proteins.

(F) Survival data for human pancreatic cancer patients from TCGA was downloaded from The Human Protein Atlas (https://www.proteinatlas.org/). Log-rank p value (Mantel-Cox test) was calculated using GraphPad Prism.

(G) The indicated human cell lines were treated with human IFNγ (1 ng/mL) for 24 h and analyzed for protein expression.

of tumor growth, there is sufficient immune-dependent IFNγ signaling to drive Ido1 expression, and this can be recapitulated at similar expression levels *in vitro* by supplementing cultured cells with IFNγ 1 ng/mL (Figures 1E and S1A). Immune cell infiltration into the tumor microenvironment is a widely observed phenomenon, but it can vary considerably from tumor to tumor.

CellPress
OPEN ACCESS

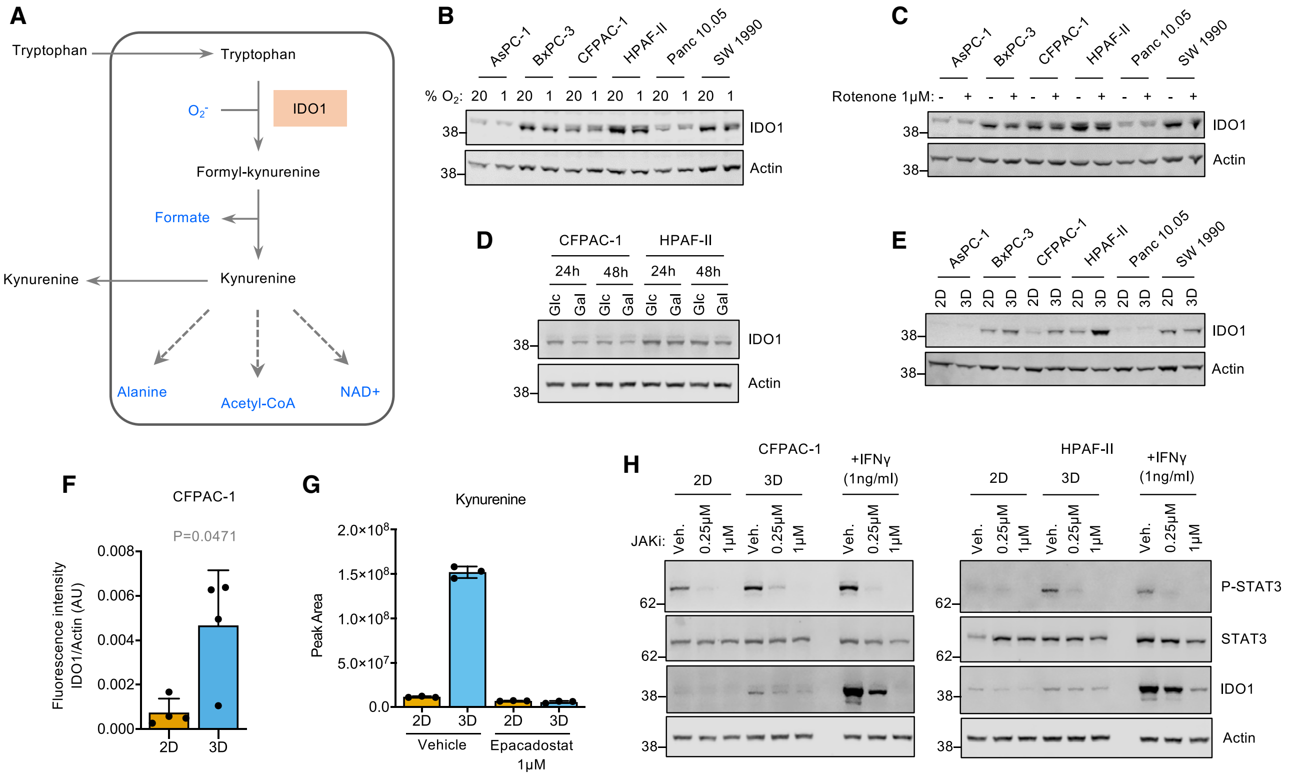

Figure 2. IDO1 is also regulated by attachment-independent growth via JAK/STAT signaling

(A) Schematic diagram of the kynurenine pathway.

(B–D) Protein expression was analyzed after 24 h of (B) culture under either normoxic (20% O_2) or hypoxic (1% O_2) conditions, (C) treatment with rotenone (1 μM) or vehicle-only control, or (D) culture in media containing either glucose (Glc) (10 mM) or galactose (Gal) (10 mM).

(E and F) Cell lines were cultured in 2D or 3D conditions for 24 h and cell lysates analyzed for protein expression, and (F) band intensity of IDO1 relative to actin (loading control) was quantified with a Li-Cor infrared scanner (data from n = 4 independent experiments, p value calculated with paired 2-sided t test, bars are SDs).

(G) CFPAC-1 cells were cultured in 2D or 3D (attachment-independent) conditions for 24 h and treated with epacadostat (1 μM) or vehicle-only control for 16 h before media (extracellular) kynurenine was analyzed by LC-MS (n = 3 biological replicates, bars are SDs).

(H) CFPAC-1 and HPAF-II cells were cultured in either 2D or 3D conditions for 24 h and then treated for 16 h with JAKi or vehicle-only control (veh.) and/or human IFNγ (1 ng/mL). Cells were then lysed and protein expression analyzed.

Genomic analysis of human PDAC tissue demonstrates that T cells and macrophages (both sources of IFNγ secretion; Gao et al., 2003) infiltrate PDAC tumors (Xu et al., 2020).

To assess IDO1 expression in human cancers, we extracted data from the metabolic gene rapid visualizer (MERAV; Shaul et al., 2016). In the pancreas, *IDO1* had a similar range of mRNA expression in healthy tissue compared to cancer cell lines grown *in vitro* (Figure S1B). However, pancreatic tumor tissue had multiple high or very high *IDO1*-expressing tumors. This trend was also observed in a variety of other tumors, particularly in the colon, breast, and cervix. This dataset shows very consistently, and in line with our observations with KPC cells, that IDO1 expression can be elevated in tumor versus healthy tissue, but that cancer cells grown under normal *in vitro* culture conditions (i.e., without IFNγ) do not recapitulate the *IDO1* expression levels seen in such tumors. These human data also highlight the potential intra-tumor variability in IDO1 expression, as seen in KPC tumors (Figures 1E and S1A). Previous work suggests that variation in cell extrinsic factors (e.g., variation in immune cell infiltration supplying IFNγ) and intrinsic factors (e.g., cyclooxygenase-2/interleukin-6 [IL-6] signaling) can contribute to this variability (Hennequart et al., 2017; Litzenburger et al., 2014). Analysis of human pancreatic cancer survival data shows that high IDO1 expression correlates with worse survival (Figure 1F).

Next, we assessed the expression of IDO1 in a panel of human pancreatic cancer cells. Similar to KPC cells, and as predicted by the MERAV data, IDO1 expression was very low or undetectable under normal culture conditions, but the addition of IFNγ (human form) consistently increased IDO1 expression (Figure 1G). Overall, these data suggest that IDO1 expression can be upregulated during tumor formation in an immunocompetent setting.

IDO1 expression can also be regulated by attachment-independent (AI) growth *in vitro*

Given the diversity of potential metabolic interactions of the kynurenine pathway (Figure 2A), we sought to investigate whether immune-independent stimuli could also affect IDO1 expression. Mitochondrial metabolism is potentially linked to the kynurenine

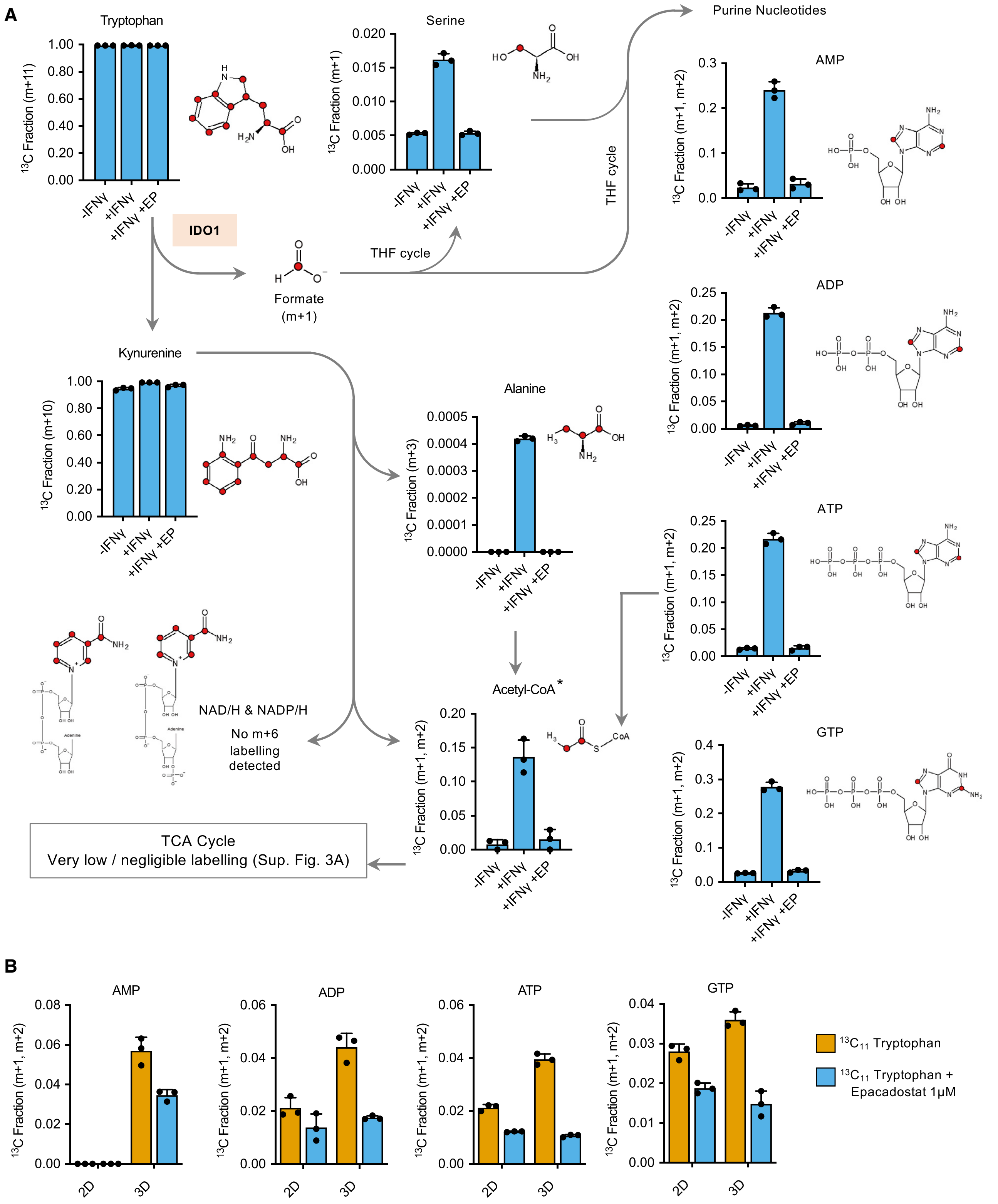

Figure 3. Tryptophan-derived one-carbon units are incorporated into serine and purine nucleotides in pancreatic cancer cells

(A and B) CFPAC-1 cells were grown with or without human IFNγ (1 ng/mL) or with IFNγ and IDO1 inhibitor epacadostat for 24 h (A), or in 2D versus 3D conditions for 24 h (B), in the presence of $^{13}C_{11}$-tryptophan. Intracellular metabolites were analyzed by LC-MS and corrected for natural background ^{13}C abundance. y axis =

(legend continued on next page)

pathway in two ways: (1) mitochondrial production of superoxide (Murphy, 2009) and (2) entry of tryptophan-derived carbons into the TCA cycle via α-ketoadipate. Exposure of PDAC cells to low oxygen or rotenone—both predicted to affect oxidative phosphorylation (OXPHOS) and potentially modulate superoxide levels—had little impact on IDO1 expression (Figures 2B and 2C). Similarly, the substitution of glucose with galactose (to promote OXPHOS) did not modulate IDO1 expression (Figure 2D). Unexpectedly, we found that transferring cells from two-dimensional (2D) monolayer culture to AI 3D growth (without any other adjustments to culture conditions) caused increased IDO1 expression in BxPC-3, CFPAC-1, HPAF-II, and SU.86.86 cells (Figures 2E, 2F, S2A, and S2B). This was accompanied by a dramatic increase in IDO1-dependent kynurenine pathway activity, as measured by kynurenine efflux, which was ablated by the IDO1 inhibitor epacadostat (Figure 2G).

AI growth stimulates IDO1 expression via Janus kinase -signal transducer and activator of transcription (JAK/STAT) signaling

While AI growth had a less dramatic impact on IDO1 expression than IFNγ, we sought to establish whether the mechanisms of these two stimuli were linked or independent. Treatment with the proteasome inhibitor MG132 (Figure S2C) or with the lysosomal inhibitor bafilomycin (Figure S2D) had no effect on IDO1 protein levels. This suggests that the increased IDO1 levels observed during AI growth were not due to changes in IDO1 degradation via proteasomal or lysosomal systems.

IFNγ mediates changes in gene expression through the activation of the JAK/STAT signaling cascade (Mojic et al., 2017). We questioned whether AI growth could also activate the JAK/STAT pathway, leading to increased IDO1 expression. We found that STAT3 phosphorylation was increased upon AI growth (Figures 1H and S2E), indicating upregulated JAK/STAT pathway activation. This appeared to be specific to STAT3, as no such increase was detected for STAT1 (Figures S2F and S2G). Upregulation of IDO1 protein levels in AI-grown cells was blocked by treatment with a JAK inhibitor (JAKi) (Figures 2H and S2E). These results suggest that whereas IFNγ stimulates JAK/STAT signaling involving both STAT1 and STAT3 activation, AI growth activates only STAT3, which results in a smaller increase in IDO1 expression. Given the ability of STAT3 to bind the *IDO1* promoter and promote *IDO1* transcription (Sun et al., 2009; Yu et al., 2014), we sought to confirm the response to AI growth at the mRNA level. qRT-PCR confirmed that AI growth increased IDO1 transcription (Figure S2H).

Tryptophan contributes one-carbon units to purine synthesis *in vitro*

During the IDO1-dependent metabolism of tryptophan through the kynurenine pathway, a number of metabolites are formed that are known to have potentially important roles in cancer metabolism (Figure 2A). To investigate the production of such metabolites from tryptophan in cancer cells expressing IDO1, we cultured cells with IFNγ in the presence of $^{13}C_{11}$-tryptophan and performed liquid chromatography mass spectrometry (LC-MS) analysis. Cells readily took up $^{13}C_{11}$-tryptophan, and over 24 h, the cellular tryptophan pool was fully labeled. As expected, we found a high fraction of labeling in kynurenine (Figures 3A and S3B). Downstream of kynurenine, a small amount of labeling was identified in alanine; however, this was a very small fraction. Tryptophan can be metabolized to acetyl-coenzyme A (CoA) via three routes: alanine, α-ketoadipate (both of which contribute to the acetyl group), or purine nucleotide synthesis (which contributes to the purine ring) (Figure S3C). While we detected substantial labeling in acetyl-CoA, very little labeling was seen in the TCA cycle (Figures S3A and S3B), illustrating that acetyl-CoA was predominantly labeled in the purine ring of coenzyme A, rather than the acetyl group that enters the TCA cycle. In addition to very low/negligible labeling in the TCA cycle, no kynurenine-dependent (m+6) labeling was detected in NAD/H or NADP/H.

During the formation of kynurenine from tryptophan, a one-carbon unit is released as formate. A potential destination for this formate is to enter the THF cycle. From there, the tryptophan-derived carbon could be used in a number of anabolic pathways, including purine nucleotide synthesis. We found clear evidence for tryptophan-derived carbons entering purine nucleotide biosynthesis (Figures 3A and S3B), indicating that tryptophan is a legitimate source of one-carbon units for the THF cycle in PDAC cells. Another THF-dependent fate for one-carbons is *de novo* serine synthesis (by combination with glycine via SHMT1/2), and we also saw increased labeling of serine from labeled tryptophan, but this was a small fraction compared to purines. Yet another THF-dependent destination for one-carbons is the re-methylation of homocysteine to methionine, which, via conversion to *S*-adenosylmethionine (SAM), can be used in methylation reactions. Interestingly, we detected a small but consistent fraction of $^{13}C_{11}$-tryptophan-dependent labeling in methionine, suggesting that tryptophan-derived carbons may also contribute to SAM synthesis and methylation reactions (Figure S3D). While the fractional labeling in kynurenine was very high under all three experimental conditions (Figures 3A and S3B), analysis of raw peak area data confirmed that the total levels of kynurenine were dramatically increased by IFNγ treatment and decreased by epacadostat, as expected (Figure S3E).

The extent of labeling seen in purines was notable, especially given that these cells were grown with ample exogenous serine, a major one-carbon source (medium contained 0.4 mM serine compared to 0.08 mM tryptophan). Labeling in serine was a much lower fraction than purines, suggesting that purine labeling occurs primarily through the direct entry of tryptophan-derived formate to the THF cycle rather than via serine synthesis.

We confirmed that AI growth was also able to stimulate the incorporation of tryptophan-derived one-carbon units into purine nucleotides, finding an increased fraction of labeling in AMP, ADP, ATP and guanosine triphosphate (GTP) in AI-grown cells

^{13}C labeled fraction for the stated isotopologues. Labeled isotopologues are defined as mass + *n* (m+*n*), where n = number of ^{13}C incorporated. The potential positions of tryptophan-derived carbons are labeled in red in the structural diagrams.
See Figure S3C for explanation of labeling in acetyl-CoA. n = 3 biological replicates, error bars are SDs.

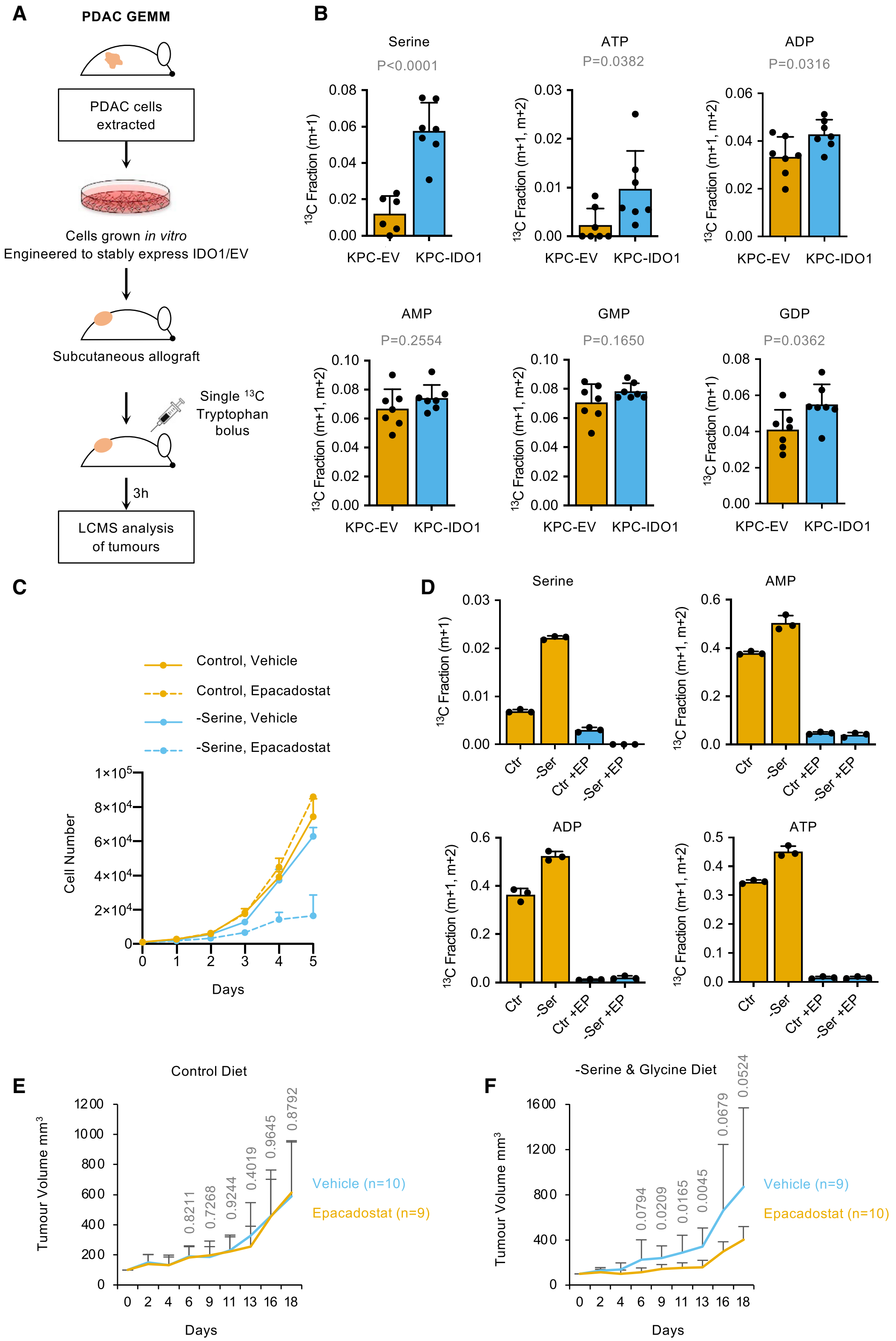

(legend on next page)

CellPress
OPEN ACCESS

(Figure 3B). This increase was far smaller than that seen in IFNγ-treated cells, reflecting the smaller increase in IDO1 expression caused by AI growth. Overall, these data clearly indicate that it is possible for IDO1-expressing PDAC cells to use tryptophan as a substantial source of one-carbon units for purine nucleotide synthesis.

Tryptophan-derived one-carbon units contribute to serine and purine synthesis in tumors and support proliferation when serine is limiting

Next, we investigated whether tryptophan could contribute one-carbon units to purine synthesis in PDAC tumors *in vivo*. Given the potential variability in IDO1 expression in autochthonous GEMM and KPC allograft tumors (taking into account data shown in Figures 1B, 1E, and S1A), and to consistently and reliably recapitulate the setting of a IDO1-expressing tumor, we engineered KPC cells to stably express IDO1. Western blot confirmed that KPC-IDO1 cells expressed lower but comparable IDO1 levels to IFNγ-treated PDAC cell lines (Figure S4A), and LC-MS analysis confirmed that KPC-IDO1 cells had upregulated tryptophan-dependent one-carbon metabolism versus KPC-EV (empty vector) cells (Figure S4B). In line with IDO1 protein expression, the extent of labeling in purines in KPC-IDO1 cells was lower but comparable with IFNγ treated PDAC cells *in vitro* (Figures 3A and S3B). These comparisons demonstrate that the KPC-IDO1 cells stably express levels of IDO1 comparable to immune-driven IDO1 expression in human cancer cells. We implanted these cells into syngeneic immunocompetent mice as subcutaneous allografts (Figure 4A). Once tumors had formed, mice received a single intraperitoneal injection of $^{13}C_{11}$-tryptophan solution, and we assessed the incorporation of tryptophan-derived carbons using LC-MS at a single time point (3 h) post-injection.

Despite the inherent difficulty of detecting labeling subsequent to just a single bolus dose of ^{13}C tracer, which is immediately further diluted by the ^{12}C pool upon injection and present only transiently, we found a small but significant increase in the labeled fraction in serine, ATP, ADP, and guanosine diphosphate (GDP) (and trends for higher labeling in AMP and guanosine monophosphate [GMP]) in the tumor tissue of IDO1-expressing versus EV controls (Figures 4B and S4C). There are different potential routes by which $^{13}C_{11}$-tryptophan-derived carbons could be metabolized into serine and purines within tumors *in vivo*: (1) tumor dependent (i.e., primarily via tumor-specific IDO1 expression), (2) systemic metabolism dependent (i.e., predominantly via IDO1 expressed in healthy tissues, which provides labeled formate or serine into the circulation, taken up by tumors), or (3) a combination of these two routes. If labeling was predominantly via systemic metabolism, then very similar levels of ^{13}C-labeling would be seen in KPC-EV and KPC-IDO1 tumors. As labeling was higher in KPC-IDO1 tumors, it strongly suggests that labeling primarily occurs via tumor-specific IDO1 expression.

To further probe the question of tumor versus systemic metabolism, we analyzed serum from $^{13}C_{11}$-tryptophan-injected mice by LC-MS and gas chromatography-MS (GC-MS). There was no difference in circulating levels of ^{13}C-labeled formate (or unlabeled formate) between mice with KPC-EV or KPC-IDO1 tumors (Figure S4D). While we were only able to detect circulating levels of two purine nucleotides (AMP and GMP), these did not show any difference in ^{13}C-labeling, and the same was seen with circulating serine (Figure S4E). These results indicate that IDO1-expressing tumors can directly metabolize tryptophan and incorporate tryptophan-derived carbons into purine nucleotides *in vivo*. Relative to *in vitro* labeling experiments, the level of serine labeling was higher *in vivo*. We speculate that compared to cell culture (in which exogenous serine is ample), there may be increased demands for *de novo* serine synthesis (e.g., to support protein synthesis).

Next, we investigated whether tryptophan could act as an alternative to serine as a one-carbon source to support tumor cell proliferation. Removal of exogenous serine alone or inhibition of IDO1 alone had little impact on the ability of IDO1-expressing KPC cells to proliferate *in vitro*. However, the combined inhibition of IDO1 and serine starvation dramatically slowed cell proliferation (Figure 4C). LC-MS analysis confirmed that the removal of exogenous serine increased the contribution of tryptophan to serine and purine synthesis in an IDO1-dependent manner (Figure 4D).

Given the combined ability of epacadostat and serine restriction to slow the proliferation of IDO-expressing cells *in vitro*, we sought to test the effect of this combination on tumor growth. A key consideration of our *in vivo* experimental design was to separate the metabolic and immunomodulatory effects of epacadostat; in other words, in an immunocompetent *in vivo* setting the impact of epacadostat on tumor growth would be a function of metabolic and immunomodulatory changes (Figure S4F). To eliminate this confounding factor and generate data specifically reflecting the metabolic impact of IDO1 inhibition *in vivo*, we used an immunocompromised mouse model in which epacadostat had no impact on tumor growth under normal conditions (Liu

Figure 4. Tryptophan-derived one-carbon units are incorporated into nucleotides in pancreatic tumors *in vivo* and can substitute for serine

(A) Schematic diagram outlining *in vivo* $^{13}C_{11}$-tryptophan tracing.

(B) KPC cell line 2 (see Figures 1E and S1A) from C57Bl6/J *Pdx1*-Cre;*Kras*$^{G12D/+}$;*Trp53*$^{R172H/+}$ mice were engineered to stably express IDO1 (KPC-IDO1) or empty vector control (KPC-EV). Cells were injected into the flanks of C57Bl6/J mice. Once tumors had formed, the mice were given a single intraperitoneal injection of 800 μL 120 mM $^{13}C_{11}$ tryptophan and harvested after 3 h. The tumor tissue was excised and analyzed by LC-MS (EV n = 7, IDO1 n = 7, p values calculated with unpaired 2-sided t test, bars are SDs). y axis = ^{13}C labeled fraction for the stated isotopologues.

(C) KPC-IDO1 cells were grown in culture medium with or without serine, with or without epacadostat (1 μM). The cell number was counted every 24 h for 5 days; n = 3 biological replicates, bars are SDs.

(D) KPC-IDO1 cells were grown in culture medium with or without serine, with or without epacadostat (1 μM) for 24 h, and intracellular metabolites were analyzed by LC-MS; n = 3 biological replicates, bars are SDs.

(E and F) KPC-IDO1 cells were injected as subcutaneous allografts into BALB/c nude mice and received vehicle or epacadostat (50 mg/kg twice per day) as oral gavage. Mice either received a control diet containing serine and glycine (E) or a matched diet lacking serine and glycine (F). Bars are SDs. p values calculated using unpaired 2-sided t test.

et al., 2010). A complication of using the immunocompromised mice is that the endogenous immunological drive for IDO1 expression is lost (as shown in Figures 1E and S1A), so to overcome this, we used KPC cells stably expressing IDO1 (and EV controls).

In mice receiving a control diet, IDO1 inhibition had no impact on tumor growth (Figure 4E), whereas in mice fed a diet lacking serine and glycine, IDO1 inhibition significantly slowed tumor growth (Figures 4F and S4G**).** We noted that the impact on tumor growth *in vivo* was not as substantial as the corresponding *in vitro* experiment (Figure 4C), possibly reflecting that serine levels can be more tightly controlled with an *in vitro* experiment.

PDAC cells excrete tryptophan-derived formate

It has been shown that cancer cells can release significant quantities of serine-derived formate (Meiser et al., 2016). We therefore questioned whether IDO1-expressing PDAC cells would release formate produced from tryptophan. After culture with $^{13}C_{11}$-tryptophan and IFNγ, labeled formate was identified by GC-MS in the spent medium from CFPAC-1 and HPAF-II cells (Figure 5A). Remarkably, the release of tryptophan-derived formate was considerably higher than serine-derived formate in CFPAC-1 cells and equivalent in HPAF-II cells. This is a striking result, given that serine is generally viewed as the dominant one-carbon source in cancer cells, and that exogenous serine levels (0.4 mM) were five times higher than tryptophan (0.08 mM). In a second efflux experiment, we equalized the tryptophan and serine concentrations (both 0.4 mM), and, after normalizing for cell number, we calculated that tryptophan-derived formate efflux with IFNγ was 13.3 fmol/cell/h in HPAF-II cells and 8.5 fmol/cell/h in CFPAC-1 cells treated with IFNγ, accounting for ~50% of total formate efflux in these cells (29.4 fmol/cell/h in HPAF-II, 14.3 fmol/cell/h in CFPAC-1) (Figure S5A). Previous studies show that other cancer cell lines have comparable total formate efflux under normal cell culture conditions (i.e., without IFNγ): HCT116 = 14–16 fmol/cell/h, IMR90 = 10 fmol/cell/h, HEK293T = 9 fmol/cell/h, and A549 = 0.9 fmol/cell/h (Ducker et al., 2016; Meiser et al., 2016). Comparison with these studies emphasizes the substantial contribution of IDO1-dependent tryptophan metabolism to the synthesis and efflux of formate by IDO1-expressing PDAC cells.

In the same experiment used for GC-MS analysis of formate efflux, we used LC-MS to analyze media samples for changes in tryptophan and kynurenine levels. This analysis shows that (with IFNγ) tryptophan consumption was 13.0 fmol/cell/h in CFPAC-1 and 32.1 fmol/cell/h in HPAF-II, but much lower without IFNγ (0.64 and 10.4 fmol/cell/h, respectively) (Figure S5B). Comparison with published levels of serine uptake (e.g., 22.5 fmol/cell/h for HCT116 and IMR90 cells [Meiser et al., 2016] and 25.2 fmol/cell/h in HEK293T cells [Ducker et al., 2016]) indicates that tryptophan uptake is generally lower than serine under normal culture conditions, but can exceed serine uptake when IFNγ is present. Notably, formate efflux was 30%–35% less than kynurenine efflux, suggesting that approximately one-third of formate generated by IDO1 was used intracellularly, with the remaining two-thirds released (Figure S5B).

Stellate cells take up cancer cell-generated tryptophan-derived formate and use it for purine synthesis

While we have previously reported on the ability of cancer cells to efflux formate (Meiser et al., 2016), with implications for cell invasive properties (Meiser et al., 2018) it is unknown whether formate efflux has any metabolic consequences for the tumor microenvironment. Spatial analysis of *Ido1* expression in the KPC tumors shown in Figure 1B demonstrated that *Ido1* expression was higher in cancer cells than stromal cells in every tumor tested (Figure S5C). Publicly available data (The Human Protein Atlas) shows several examples of human PDAC tumors with strong IDO1 expression, and that IDO1 is primarily confined to cancer cells rather than stromal cells (Figure S5D). Given the dense stromal component of pancreatic tumors, we speculated that exogenous formate could affect stromal cell metabolism.

To directly test the ability of pancreatic stellate cells to take up and use exogenous formate, we cultured immortalized mouse pancreatic stellate cells (ImPSCs) in media supplemented with $^{13}C_1$-formate. LC-MS revealed that stellate cells were capable of consuming extracellular formate and incorporating its single carbon into purines (Figure S5E). To assess whether tryptophan-derived formate produced within PDAC cells could be used in the same way, we used two techniques: (1) conditioned media transfer and (2) direct co-culture (Figure 5B). To condition the medium, PDAC cells were grown in medium containing $^{13}C_{11}$-tryptophan, and conditioned medium was collected after 24 h. Filtered medium was then transferred onto stellate cells before analysis of the stellate cells by LC-MS. Labeling of purine nucleotides and serine was detected in stellate cells given conditioned medium from IDO1-expressing (+IFNγ) PDAC cells (Figure 5C). Labeling was prevented when the PDAC cells were treated with epacadostat during the conditioning process. Critically, labeling was unaffected when epacadostat was added after conditioning (i.e., while stellate cells were grown with conditioned medium), indicating that IDO1 activity in the PDAC cells, not the stellate cells, is critical for stellate cell formate utilization. This was also found when the experiment was repeated with ImPSC #2 cells (Figure S5F). To further confirm these findings, we performed direct co-culture assays. CFPAC-1 cells were co-cultured for 24 h with ImPSC cells engineered to ectopically express GFP. The co-culture medium contained $^{13}C_{11}$-tryptophan and IFNγ. The ImPSC-GFP cells were then separated from the PDAC cells by fluorescence-activated cell sorting (FACS) and subjected to LC-MS analysis. Labeling of purine nucleotides was evident in stellate cells co-cultured with PDAC cells in the presence of IFNγ (Figure 5D). However, with this more technically challenging method, the labeled fractions were generally smaller and labeling in serine was very similar to background levels. Importantly, labeling was lower when IDO1 levels were low (−IFNγ) or IDO1 was inhibited by treatment with epacadostat (Figure 5D). These results show that pancreatic stellate cells can consume and metabolize tryptophan-derived formate that has been released by PDAC cells in an IDO1-dependent manner (Figure 5E).

DISCUSSION

While serine is thought to be a dominant source of one-carbon units, recent work has shown that other amino acids and

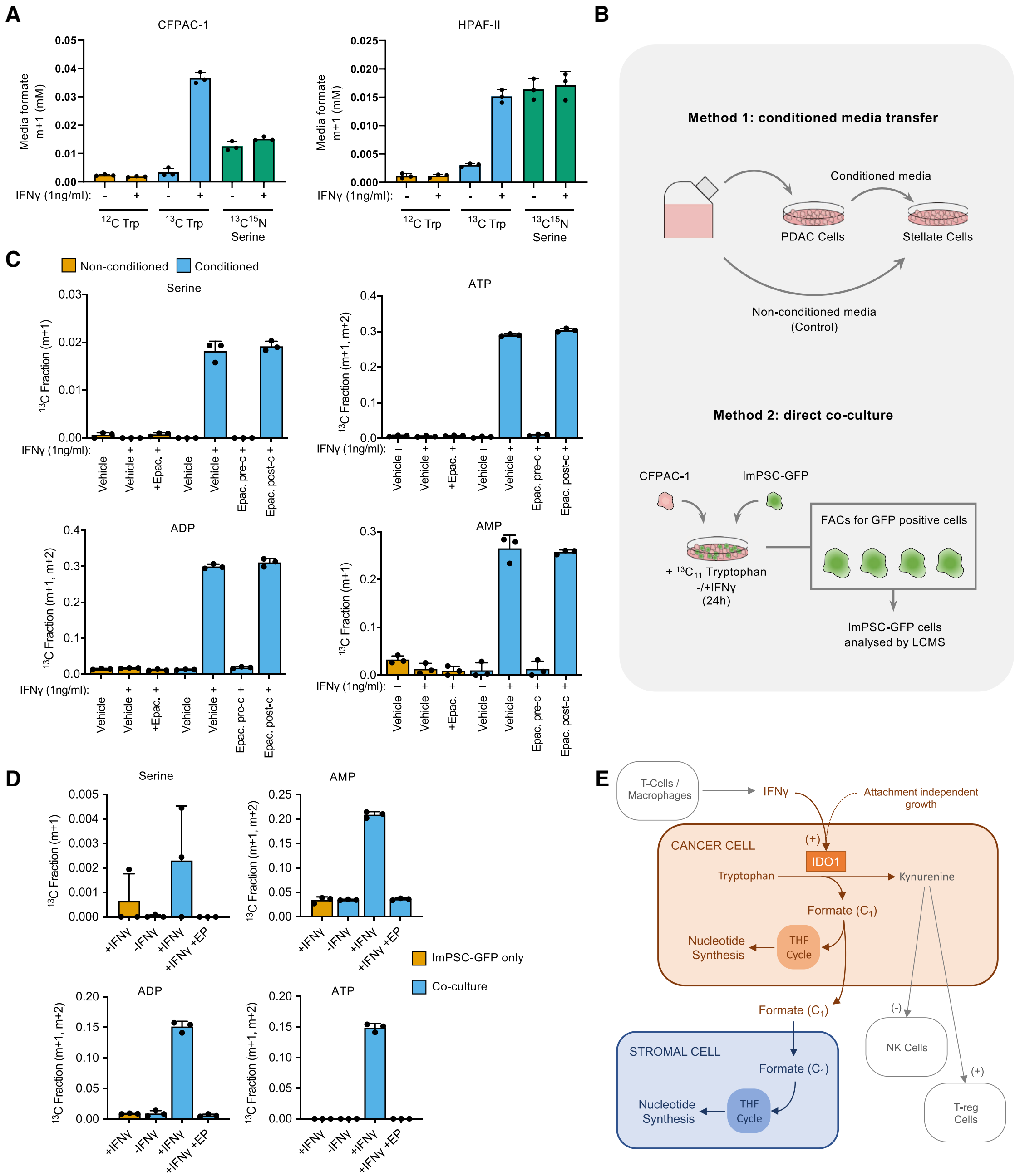

Figure 5. PDAC cells release tryptophan-derived formate, which is consumed by pancreatic stellate cells and incorporated into nucleotides

(A) CFPAC-1 and HPAF-II cells were cultured in 3D for 4 days and then treated with IFNγ (1 ng/mL) or vehicle-only control in the presence of either unlabeled (^{12}C), $^{13}C_{11}$-tryptophan 0.08 mM, or $^{13}C_3{}^{15}N_1$-serine 0.4 mM for 24 h. Media samples were analyzed for extracellular formate using GC-MS; n = 3 biological replicates, bars are SDs. y axis = ^{13}C labeled fraction for the stated isotopologues.

(B) Schematic diagram of the experimental approaches used in (C) and (D).

(C) CFPAC-1 cells were treated with vehicle-only control or human IFNγ (1 ng/mL) and epacadostat (Epac., 1 μM) or vehicle-only control in the presence of unlabeled (^{12}C) or $^{13}C_{11}$ tryptophan. Conditioned media was collected after 24 h and ImPSCs (immortalized pancreatic stellate cells) were cultured in this media or in non-conditioned treatment-matched media. After 24 h, intracellular metabolites were analyzed by LC-MS; n = 3 biological replicates, bars are SDs.

(D), ImPSC-GFP cells were cultured for 24 h as a monoculture or in co-culture with CFPAC-1 cells. Cells were then treated with vehicle-only control or human IFNγ (1 ng/mL) and epacadostat (1 μM) or vehicle-only control in the presence of $^{13}C_{11}$ tryptophan for 24 h. Cells were then trypsinized and sorted using FACS for GFP$^+$ cells and intracellular metabolites were analyzed by LC-MS; n = 3 biological replicates, bars are SDS.

I Illustration of the proposed model.

formaldehyde (Burgos-Barragan et al., 2017) can contribute to the THF cycle in ways that can affect cancer therapy (Kanarek et al., 2018). Improved understanding of which nutrients and pathways feed the THF cycle will enhance our ability to deploy emerging anti-cancer strategies such as dietary serine withdrawal or serine synthesis inhibition, as well as traditional antifolate therapies. The kynurenine pathway has been studied intensively for its ability to modulate immune cell behavior. In particular, IDO1 inhibitors have been aggressively explored as anti-cancer immunotherapies. Results from clinical trials have so far been mixed, leading to the wider activities of IDO1 being described as a "black box," and calls for improved mechanistic understanding of this enzyme (Garber, 2018). Given the significant clinical interest in IDO1, its known role as a metabolic enzyme, and the widespread engagement in cancer metabolism research, it is surprising that to date little effort has been made to interrogate the metabolic functions of this enzyme. This is especially relevant given the potential importance of kynurenine pathway-derived metabolites for cancer cell metabolism.

While serine is considered the primary source of one-carbon units in cancer cells, the present study highlights that in IDO1-expressing cancer cells, tryptophan is potentially a major alternative source of one-carbons for the THF cycle. Multiple studies have reported on the ability of a serine- and glycine-deficient diet to slow tumor growth in preclinical cancer models (Baksh et al., 2020; LeBoeuf et al., 2020; Maddocks et al., 2013, 2017; Muthusamy et al., 2020). Interestingly, KPC models resist dietary serine and glycine restriction (Maddocks et al., 2017). Previously we attributed this solely to the upregulation of *de novo* serine synthesis; the present study suggests that utilization of tryptophan can substitute for serine as a one-carbon donor, a process that can be targeted with IDO1 inhibitors such as epacadostat. The ability of KPC tumors to eventually grow despite serine restriction and epacadostat treatment suggests that *de novo* serine synthesis does contribute to resistance, but that this takes longer to initiate than switching to tryptophan.

Overall, these results shed increased light on IDO function, and suggest that IDO1 could have an important role beyond immune regulation, with the potential to influence one-carbon metabolism in cancer and stromal cells. We show that in IDO1-expressing tumors, tryptophan is a legitimate one-carbon source for the THF cycle. Our data also provide a mechanistic explanation as to why tryptophan has been reported as one of the most highly depleted interstitial nutrients in PDAC (Sullivan et al., 2019a). Furthermore, our data show that biological context is critical for the study of IDO1 function, with standard cell culture conditions being a poor model. While this study has focused on pancreatic cancer, human IDO1 expression data suggest that our findings are relevant across a range of other human cancers, particularly colon, breast, cervix, and lung.

Limitations

Further exploration of the link between attachment-independent growth and STAT3/IDO1 activation (e.g., in the context of metastasis formation) would help to better define the biological relevance of this phenotype to cancer. Our data also illustrate a wide variability in the expression of IDO1 *in vivo*. While the literature suggests that this could be accounted for by cell-intrinsic and -extrinsic factors, a more thorough exploration of what dictates tumor IDO1 expression would be valuable—for example, in helping to stratify treatment with IDO1 inhibitors. While our short-term *in vivo* ^{13}C-tryptophan experiment did show a change in tumor tryptophan metabolism due to IDO1 expression, a longer-term analysis (e.g., using constant infusion) would be required to more accurately quantify the scale of this effect. While our *in vivo* tumor growth experiment did show cooperation between dietary serine and glycine restriction and epacadostat treatment, this was far less dramatic than the corresponding *in vitro* data. Further tumor growth assays are required to more fully characterize this effect—for example, using immunocompetent models in which the combined metabolic and immunomodulatory effects of IDO1 inhibition are taken into account.

STAR★METHODS

Detailed methods are provided in the online version of this paper and include the following:

- KEY RESOURSES TABLE
- RESOURCE AVAILABILITY
  - Lead contact
  - Materials availability
  - Data and code availability
- EXPERIMENTAL MODEL AND SUBJECT DETAILS
  - Cell culture
  - Mice
- METHOD DETAILS
  - Extraction of ImPSC cell lines
  - Production of KPC-EV and KPC-IDO1 cell lines
  - Hypoxia experiments
  - Attachment independent 3D growth experiments
  - Conditioned medium experiments
  - Co-culture experiments
  - RNA extraction, reverse transcription, and expression analysis
  - RNA-Scope *in situ* hybridization and Immunohistochemistry
  - Survival analysis
  - Western blotting
  - *In vivo* models
  - LCMS for steady-state metabolite measurements
  - GCMS for formate analysis
  - Carbon-13 (^{13}C) labeling of metabolites
- QUANTIFICATION AND STATISTICAL ANALYSIS

SUPPLEMENTAL INFORMATION

Supplemental information can be found online at https://doi.org/10.1016/j.molcel.2021.03.019.

ACKNOWLEDGMENTS

We thank Biological Services facility staff at the Cancer Research UK Beatson Institute, funded by Cancer Research UK (A18076 and A17196), and Colin Nixon and Gemma Thomson for assistance with immunohistochemistry (IHC) and *in situ* hybridization (ISH). We thank J.P. Morton and S.A. Karim for the KPC cell lines, J.J. Kamphorst for the ImPSC #1 cell line, and O.J. Sansom and D.F. Vincent for providing the Pdgfra$^{tm11(EGFP)Sor}$ mice and assisting

CellPress
OPEN ACCESS

with the extraction of ImPSC #2 and #3 cell lines. A.C.N., O.D.K.M., A.H.U., and T.Z. were funded by a Cancer Research UK Career Development Fellowship awarded to O.D.K.M. (C53309/A19702). M.F. was funded by an EMBO Long-Term Fellowship (EMBO ALTF 276-2019). K.B. and D.A. were core funded by Cancer Research UK (A17196 and A29799). M.P. and A.V. were funded by Cancer Research UK.

AUTHOR CONTRIBUTIONS

A.C.N. designed and performed the cell culture experiments, western blots, analysis and interpretation of LC-MS and other data, and co-wrote the manuscript. M.F. performed the cell culture experiments, western blots, and analysis of The Cancer Genome Atlas (TCGA) data. A.H.U. and T.Z. performed the mass spectrometry and analysis of the LC-MS data. D.A. performed and analyzed data for the *in vivo* experiments. M.P. and A.V. contributed to designing and performing the GC-MS analysis of the formate levels. K.B. contributed to designing, supervising, and analyzing the *in vivo* experiments. O.D.K.M. conceived and designed the study, analyzed and interpreted the data, and co-wrote the manuscript. All of the authors edited and approved the final version of the manuscript.

DECLARATION OF INTERESTS

O.D.K.M. contributed to CRUK Cancer Research Technology filing of UK patent application no. 1609441.9, relating to dietary modulation of amino acids, and is a co-founder, shareholder, and advisor of Faeth Therapeutics. The other authors declare no competing interests.

Received: January 13, 2020
Revised: December 15, 2020
Accepted: March 12, 2021
Published: April 7, 2021

REFERENCES

Auciello, F.R., Bulusu, V., Oon, C., Tait-Mulder, J., Berry, M., Bhattacharyya, S., Tumanov, S., Allen-Petersen, B.L., Link, J., Kendsersky, N.D., et al. (2019). A Stromal Lysolipid-Autotaxin Signaling Axis Promotes Pancreatic Tumor Progression. Cancer Discov. *9*, 617–627.

Baksh, S.C., Todorova, P.K., Gur-Cohen, S., Hurwitz, B., Ge, Y., Novak, J.S.S., Tierney, M.T., Dela Cruz-Racelis, J., Fuchs, E., and Finley, L.W.S. (2020). Extracellular serine controls epidermal stem cell fate and tumour initiation. Nat. Cell Biol. *22*, 779–790.

Burgos-Barragan, G., Wit, N., Meiser, J., Dingler, F.A., Pietzke, M., Mulderrig, L., Pontel, L.B., Rosado, I.V., Brewer, T.F., Cordell, R.L., et al. (2017). Mammals divert endogenous genotoxic formaldehyde into one-carbon metabolism. Nature *548*, 549–554.

Ducker, G.S., Chen, L., Morscher, R.J., Ghergurovich, J.M., Esposito, M., Teng, X., Kang, Y., and Rabinowitz, J.D. (2016). Reversal of Cytosolic One-Carbon Flux Compensates for Loss of the Mitochondrial Folate Pathway. Cell Metab. *23*, 1140–1153.

Gao, Y., Yang, W., Pan, M., Scully, E., Girardi, M., Augenlicht, L.H., Craft, J., and Yin, Z. (2003). Gamma delta T cells provide an early source of interferon gamma in tumor immunity. J. Exp. Med. *198*, 433–442.

Garber, K. (2018). A new cancer immunotherapy suffers a setback. Science *360*, 588.

Hamilton, T.G., Klinghoffer, R.A., Corrin, P.D., and Soriano, P. (2003). Evolutionary divergence of platelet-derived growth factor alpha receptor signaling mechanisms. Mol. Cell. Biol. *23*, 4013–4025.

Hanahan, D., and Weinberg, R.A. (2011). Hallmarks of cancer: the next generation. Cell *144*, 646–674.

Hennequart, M., Pilotte, L., Cane, S., Hoffmann, D., Stroobant, V., Plaen, E., and Van den Eynde, B.J. (2017). Constitutive IDO1 Expression in Human Tumors Is Driven by Cyclooxygenase-2 and Mediates Intrinsic Immune Resistance. Cancer Immunol. Res. *5*, 695–709.

Hingorani, S.R., Wang, L., Multani, A.S., Combs, C., Deramaudt, T.B., Hruban, R.H., Rustgi, A.K., Chang, S., and Tuveson, D.A. (2005). Trp53R172H and KrasG12D cooperate to promote chromosomal instability and widely metastatic pancreatic ductal adenocarcinoma in mice. Cancer Cell *7*, 469–483.

Hornyák, L., Dobos, N., Koncz, G., Karányi, Z., Páll, D., Szabó, Z., Halmos, G., and Székvölgyi, L. (2018). The Role of Indoleamine-2,3-Dioxygenase in Cancer Development, Diagnostics, and Therapy. Front. Immunol. *9*, 151.

Jain, M., Nilsson, R., Sharma, S., Madhusudhan, N., Kitami, T., Souza, A.L., Kafri, R., Kirschner, M.W., Clish, C.B., and Mootha, V.K. (2012). Metabolite profiling identifies a key role for glycine in rapid cancer cell proliferation. Science *336*, 1040–1044.

Kanarek, N., Keys, H.R., Cantor, J.R., Lewis, C.A., Chan, S.H., Kunchok, T., Abu-Remaileh, M., Freinkman, E., Schweitzer, L.D., and Sabatini, D.M. (2018). Histidine catabolism is a major determinant of methotrexate sensitivity. Nature *559*, 632–636.

Katz, J.B., Muller, A.J., and Prendergast, G.C. (2008). Indoleamine 2,3-dioxygenase in T-cell tolerance and tumoral immune escape. Immunol. Rev. *222*, 206–221.

Kim, D., Fiske, B.P., Birsoy, K., Freinkman, E., Kami, K., Possemato, R.L., Chudnovsky, Y., Pacold, M.E., Chen, W.W., Cantor, J.R., et al. (2015). SHMT2 drives glioma cell survival in ischaemia but imposes a dependence on glycine clearance. Nature *520*, 363–367.

Labuschagne, C.F., van den Broek, N.J., Mackay, G.M., Vousden, K.H., and Maddocks, O.D. (2014). Serine, but not glycine, supports one-carbon metabolism and proliferation of cancer cells. Cell Rep. *7*, 1248–1258.

LeBoeuf, S.E., Wu, W.L., Karakousi, T.R., Karadal, B., Jackson, S.R., Davidson, S.M., Wong, K.K., Koralov, S.B., Sayin, V.I., and Papagiannakopoulos, T. (2020). Activation of Oxidative Stress Response in Cancer Generates a Druggable Dependency on Exogenous Non-essential Amino Acids. Cell Metab. *31*, 339–350.e4.

Litzenburger, U.M., Opitz, C.A., Sahm, F., Rauschenbach, K.J., Trump, S., Winter, M., Ott, M., Ochs, K., Lutz, C., Liu, X., et al. (2014). Constitutive IDO expression in human cancer is sustained by an autocrine signaling loop involving IL-6, STAT3 and the AHR. Oncotarget *5*, 1038–1051.

Liu, X., Shin, N., Koblish, H.K., Yang, G., Wang, Q., Wang, K., Leffet, L., Hansbury, M.J., Thomas, B., Rupar, M., et al. (2010). Selective inhibition of IDO1 effectively regulates mediators of antitumor immunity. Blood *115*, 3520–3530.

Locasale, J.W., Grassian, A.R., Melman, T., Lyssiotis, C.A., Mattaini, K.R., Bass, A.J., Heffron, G., Metallo, C.M., Muranen, T., Sharfi, H., et al. (2011). Phosphoglycerate dehydrogenase diverts glycolytic flux and contributes to oncogenesis. Nat. Genet. *43*, 869–874.

Maddocks, O.D., Berkers, C.R., Mason, S.M., Zheng, L., Blyth, K., Gottlieb, E., and Vousden, K.H. (2013). Serine starvation induces stress and p53-dependent metabolic remodelling in cancer cells. Nature *493*, 542–546.

Maddocks, O.D.K., Athineos, D., Cheung, E.C., Lee, P., Zhang, T., van den Broek, N.J.F., Mackay, G.M., Labuschagne, C.F., Gay, D., Kruiswijk, F., et al. (2017). Modulating the therapeutic response of tumours to dietary serine and glycine starvation. Nature *544*, 372–376.

Meiser, J., Tumanov, S., Maddocks, O., Labuschagne, C.F., Athineos, D., Van Den Broek, N., Mackay, G.M., Gottlieb, E., Blyth, K., Vousden, K., et al. (2016). Serine one-carbon catabolism with formate overflow. Sci. Adv. *2*, e1601273.

Meiser, J., Schuster, A., Pietzke, M., Vande Voorde, J., Athineos, D., Oizel, K., Burgos-Barragan, G., Wit, N., Dhayade, S., Morton, J.P., et al. (2018). Increased formate overflow is a hallmark of oxidative cancer. Nat. Commun. *9*, 1368.

Mezrich, J.D., Fechner, J.H., Zhang, X., Johnson, B.P., Burlingham, W.J., and Bradfield, C.A. (2010). An interaction between kynurenine and the aryl hydrocarbon receptor can generate regulatory T cells. J. Immunol. *185*, 3190–3198.

Mojic, M., Takeda, K., and Hayakawa, Y. (2017). The Dark Side of IFN-γ: Its Role in Promoting Cancer Immunoevasion. Int. J. Mol. Sci. *19*, 89.

Morton, J.P., Timpson, P., Karim, S.A., Ridgway, R.A., Athineos, D., Doyle, B., Jamieson, N.B., Oien, K.A., Lowy, A.M., Brunton, V.G., et al. (2010). Mutant

p53 drives metastasis and overcomes growth arrest/senescence in pancreatic cancer. Proc. Natl. Acad. Sci. USA *107*, 246–251.

Munn, D.H., Sharma, M.D., Baban, B., Harding, H.P., Zhang, Y., Ron, D., and Mellor, A.L. (2005). GCN2 kinase in T cells mediates proliferative arrest and anergy induction in response to indoleamine 2,3-dioxygenase. Immunity *22*, 633–642.

Murphy, M.P. (2009). How mitochondria produce reactive oxygen species. Biochem. J. *417*, 1–13.

Muthusamy, T., Cordes, T., Handzlik, M.K., You, L., Lim, E.W., Gengatharan, J., Pinto, A.F.M., Badur, M.G., Kolar, M.J., Wallace, M., et al. (2020). Serine restriction alters sphingolipid diversity to constrain tumour growth. Nature *586*, 790–795.

Newman, A.C., and Maddocks, O.D.K. (2017a). One-carbon metabolism in cancer. Br. J. Cancer *116*, 1499–1504.

Newman, A.C., and Maddocks, O.D.K. (2017b). Serine and Functional Metabolites in Cancer. Trends Cell Biol. *27*, 645–657.

Pacold, M.E., Brimacombe, K.R., Chan, S.H., Rohde, J.M., Lewis, C.A., Swier, L.J., Possemato, R., Chen, W.W., Sullivan, L.B., Fiske, B.P., et al. (2016). A PHGDH inhibitor reveals coordination of serine synthesis and one-carbon unit fate. Nat. Chem. Biol. *12*, 452–458.

Possemato, R., Marks, K.M., Shaul, Y.D., Pacold, M.E., Kim, D., Birsoy, K., Sethumadhavan, S., Woo, H.K., Jang, H.G., Jha, A.K., et al. (2011). Functional genomics reveal that the serine synthesis pathway is essential in breast cancer. Nature *476*, 346–350.

Prendergast, G.C. (2008). Immune escape as a fundamental trait of cancer: focus on IDO. Oncogene *27*, 3889–3900.

Shaul, Y.D., Yuan, B., Thiru, P., Nutter-Upham, A., McCallum, S., Lanzkron, C., Bell, G.W., and Sabatini, D.M. (2016). MERAV: a tool for comparing gene expression across human tissues and cell types. Nucleic Acids Res. *44* (D1), D560–D566.

Sonner, J.K., Deumelandt, K., Ott, M., Thomé, C.M., Rauschenbach, K.J., Schulz, S., Munteanu, B., Mohapatra, S., Adam, I., Hofer, A.C., et al. (2016). The stress kinase GCN2 does not mediate suppression of antitumor T cell responses by tryptophan catabolism in experimental melanomas. OncoImmunology *5*, e1240858.

Sousa, C.M., Biancur, D.E., Wang, X., Halbrook, C.J., Sherman, M.H., Zhang, L., Kremer, D., Hwang, R.F., Witkiewicz, A.K., Ying, H., et al. (2016). Pancreatic stellate cells support tumour metabolism through autophagic alanine secretion. Nature *536*, 479–483.

Sullivan, M.R., Danai, L.V., Lewis, C.A., Chan, S.H., Gui, D.Y., Kunchok, T., Dennstedt, E.A., Vander Heiden, M.G., and Muir, A. (2019a). Quantification of microenvironmental metabolites in murine cancers reveals determinants of tumor nutrient availability. eLife *8*, e44235.

Sullivan, M.R., Mattaini, K.R., Dennstedt, E.A., Nguyen, A.A., Sivanand, S., Reilly, M.F., Meeth, K., Muir, A., Darnell, A.M., Bosenberg, M.W., et al. (2019b). Increased Serine Synthesis Provides an Advantage for Tumors Arising in Tissues Where Serine Levels Are Limiting. Cell Metab. *29*, 1410–1421.e4.

Sun, Y., Chin, Y.E., Weisiger, E., Malter, C., Tawara, I., Toubai, T., Gatza, E., Mascagni, P., Dinarello, C.A., and Reddy, P. (2009). Cutting edge: negative regulation of dendritic cells through acetylation of the nonhistone protein STAT-3. J. Immunol. *182*, 5899–5903.

Terness, P., Bauer, T.M., Röse, L., Dufter, C., Watzlik, A., Simon, H., and Opelz, G. (2002). Inhibition of allogeneic T cell proliferation by indoleamine 2,3-dioxygenase-expressing dendritic cells: mediation of suppression by tryptophan metabolites. J. Exp. Med. *196*, 447–457.

Xu, C., Sui, S., Shang, Y., Yu, Z., Han, J., Zhang, G., Ntim, M., Hu, M., Gong, P., Chen, H., and Zhang, X. (2020). The landscape of immune cell infiltration and its clinical implications of pancreatic ductal adenocarcinoma. J. Adv. Res. *24*, 139–148.

Yu, J., Wang, Y., Yan, F., Zhang, P., Li, H., Zhao, H., Yan, C., Yan, F., and Ren, X. (2014). Noncanonical NF-κB activation mediates STAT3-stimulated IDO upregulation in myeloid-derived suppressor cells in breast cancer. J. Immunol. *193*, 2574–2586.

Yu, C.P., Fu, S.F., Chen, X., Ye, J., Ye, Y., Kong, L.D., and Zhu, Z. (2018). The Clinicopathological and Prognostic Significance of IDO1 Expression in Human Solid Tumors: Evidence from a Systematic Review and Meta-Analysis. Cell. Physiol. Biochem. *49*, 134–143.

CellPress
OPEN ACCESS

STAR★METHODS

KEY RESOURSES TABLE

REAGENT or RESOURCE	SOURCE	IDENTIFIER
Antibodies		
Rabbit polyclonal anti-IDO1 [human]	Sigma	Cat#HPA023072
Rabbit monoclonal anti-IDO1 [human] (D5J4E)	CST	Cat#86630
Rabbit monoclonal anti-IDO1 [mouse] (D8W5E)	CST	Cat#51851
Mouse monoclonal anti-Actin (C4)	Millipore	Cat#MAB1501
Rabbit monoclonal anti-phospho-STAT1 (58D6)	CST	Cat#9167
Mouse monoclonal anti-STAT1 (9H2)	CST	Cat#9176
Rabbit monoclonal anti-phospho-STAT3 (D3A7)	CST	Cat#9145
Mouse monoclonal anti-STAT3 (124H6)	CST	Cat#9139
Mouse monoclonal anti-p62 (Clone 3)	BD Biosciences	Cat#610832
IRDye® 680RD Donkey anti-Rabbit IgG Secondary Antibody	LI-COR	Cat#925-68073
IRDye® 800CW Donkey anti-Mouse IgG Secondary Antibody	LI-COR	Cat#925-32212
Mouse monoclonal anti-actin, α-smooth muscle (α-SMA; clone 1A4)	Sigma	Cat#A2547
Rabbit polyclonal anti-EpCAM	Abcam	Cat#ab71916
Chemicals, peptides, and recombinant proteins		
Human IFNγ	GIBCO	Cat#PHC4031
Mouse IFNγ	GIBCO	Cat#PMC4033
Rotenone	Sigma-Aldrich	Cat#R8875
Epacadostat	Cayman Chemical	Cat#19875
JAK Inhibitor I (JAKi)	Cayman Chemical	Cat#15146
MG-132	Calbiochem	Cat#474790-1MG
Bafilomycin A1	Sigma-Aldrich	Cat# B1793-2UG
REVERT Total Protein Stain	LI-COR	Cat#926-11010
FORMIC ACID (13C, 99%) (< 5% H2O)	Cambridge Isotope Laboratories, Inc.	Cat# CLM-1284-PK
L-Tryptophan (13C11, 99%)	Cambridge Isotope Laboratories, Inc.	Cat# CLM-4290-H-PK
L-Serine (13C3, 99%; 15N, 99%)	Cambridge Isotope Laboratories, Inc.	Cat#CNLM-474-H-PK
Deposited data		
Uncropped western blots & LCMS isotopologue data Maddocks, Oliver (2021), "Immune regulated IDO1-dependent tryptophan metabolism is source of one-carbon units for pancreatic cancer and stellate cells. Newman et al, Feb 2021.," Mendeley Data, V1, https://doi.org/10.17632/2wwd7hrxs3.1	This paper	https://doi.org/10.17632/2wwd7hrxs3.1
Uncropped western blots & LCMS isotopologue data will also be available from the University of Glasgow	https://www.gla.ac.uk/researchinstitutes/cancersciences/staff/olivermaddocks/#researchdatasets	N/A

(*Continued on next page*)

Continued		
REAGENT or RESOURCE	SOURCE	IDENTIFIER
Figure S1B: Data extracted from the MERAV database showing the relative abundance of IDO1 mRNA from microarray data across a range of human cell lines and tumor tissue.	Shaul et al., 2016	N/A
Figure 1F: Survival data for human pancreatic cancer patients from TCGA was downloaded from the Human Protein Atlas	https://www.proteinatlas.org/	N/A
Experimental models: cell lines		
Human: CFPAC-1	ATCC (LGC Standards)	CRL-1918
Human: HPAF-II	ATCC (LGC Standards)	CRL-1997
Human: AsPC-1	ATCC (LGC Standards)	CRL-1682
Human: BxPC-3	ATCC (LGC Standards)	CRL-1687
Human: Panc 10.05	ATCC (LGC Standards)	CRL-2547
Human: SW 1990	ATCC (LGC Standards)	CRL-2172
Human: SU.86.86	ATCC (LGC Standards)	CRL-1837
Mouse: ImPSC #1	Jurre Kamhorst Lab (Cancer Research UK Beatson Institute)	N/A
Mouse: ImPSC #2	This paper	N/A
Mouse: ImPSC #3	This paper	N/A
Mouse: ImPSC #1–PB-GFP	This paper	N/A
Mouse: KPC A mixed background	Jennifer Morton Lab (Cancer Research UK Beatson Institute)	N/A
Mouse: KPC pure C57BL/J background #1	Jennifer Morton Lab (Cancer Research UK Beatson Institute)	N/A
Mouse: KPC pure C57BL/J background #2	Jennifer Morton Lab (Cancer Research UK Beatson Institute)	N/A
Mouse: KPC pure C57BL/J background #3	Jennifer Morton Lab (Cancer Research UK Beatson Institute)	N/A
Experimental models: organisms/strains		
Mouse: Crl:CD1-Foxn1^{nu} (CD-1 Nude)	Charles River	Strain 086
Mouse: C57Bl6/J	Charles River	Strain 632
Mouse: CAnN.Cg-Foxn1^{nu}/Crl (BALB/c Nude)	Charles River	Strain 194
Mouse: Pdgfra$^{tm11(EGFP)Sor}$	The Jackson Laboratory	JAX #007669{Hamilton, 2003 #28}
Recombinant DNA		
Super piggyBac Transposase expression vector	Cambridge Bioscience	Cat#PB210PA-1
PB-GFP PB-CMV-MCS-EF1-GreenPuro cDNA cloning and expression vector	Cambridge Bioscience	Cat#PB513B-1
PB-RFP PB-CMV-MCS-EF1-RedPuro cDNA cloning and expression vector	Cambridge Bioscience	Cat#PB514B-2
PB-RFP PB-CMV-IDO1-EF1-RedPuro	This paper	N/A
RNAScope Probe: Ido1	Advanced Cell Diagnostics	315978
RNAScope Probe: Positive control PPIB (2.5 LS)	Advanced Cell Diagnostics	313918
Software and algorithms		
GraphPad Prism v8 & v9	GraphPad software	N/A
MZMine 2.10	MZMine	N/A
Image Studio version 5.2	LI-COR	N/A
MassHunter Quantitative Data Analysis version B.06.00	Agilent technologies	N/A

CellPress
OPEN ACCESS

RESOURCE AVAILABILITY

Lead contact

Further information and requests for resources and reagents should be directed to and will be fulfilled by the Lead Contact, Oliver D. K. Maddocks (Oliver.Maddocks@glasgow.ac.uk)

Materials availability

All unique / stable reagents generated in this study are available from the Lead Contact and may require a completed Materials Transfer Agreement.

Data and code availability

The datasets generated in this study will be made publicly available at: https://www.gla.ac.uk/researchinstitutes/cancersciences/staff/olivermaddocks/#researchdatasets. Uncropped western blots and related data is available at: http://doi.org/10.17632/2wwd7hrxs3.1

EXPERIMENTAL MODEL AND SUBJECT DETAILS

Cell culture

All the cell lines used in this study were cultured at 37°C in 5% CO_2 in a humidified incubator. Human cell lines were authenticated using Promega GenePrint 10 and tested for mycoplasma using Mycoalert (Lonza). AsPC-1 (female), BxPC-3 (female), CFPAC-1 (male), HPAF-II (male), Panc 10.05 (male) & SW 1990 (male) cells were cultured in RPMI (Invitrogen, 31870025) supplemented with 10% FBS, 1% penicillin-streptomycin, 0.2% amphotericin B and glutamine (2mM). Mouse ImPSC and KPC cell lines were cultured in DMEM (Invitrogen, 21969035) supplemented with 10% FBS, 1% penicillin-streptomycin, 0.2% amphotericin B and glutamine (2mM). KPC lines were a gift from Jennifer Morton and Saadia Karim (Ximbio, 153474), and were isolated from the tumors of *Pdx1*-cre;LSL-*Kras*$^{G12D/+}$;LSL-*Trp53*$^{R172H/+}$ mice either with a mixed or pure C57Bl6/J background. KPC-IDO1 & KPC-EV cell lines were made from pure C57Bl6/J KPC cells using the PiggyBac transposon system. ImPSC #1 lines were a gift from Jurre Kamphorst(Auciello et al., 2019). ImPSC #2 & #3 lines were isolated from Pdgfra$^{tm11(EGFP)Sor}$ mice(Hamilton et al., 2003) (JAX stock #007669).

Mice

All *in vivo* work was carried out in compliance with the Animals (Scientific Procedures) Act 1986 and the EU Directive 2010 (PPLs 60/4181, 70/8645, 70/8468 and 70/8646) and was sanctioned by the local ethical review process (University of Glasgow). *Mus musculus* cohorts were housed in a barrier facility proactive in environmental enrichment and maintained on a normal chow diet *ad libitum*. Mice were genotyped by Transnetyx (Cordova, Tennessee, USA). The LSL-Kras^{G12D}, Pdx1-cre;LSL-Kras$^{G12D/+}$;Trp53$^{fl/+}$ and Pdx1-cre;LSL-Kras$^{G12D/+}$;LSL-Trp53$^{R172H/+}$ models/alleles have been described previously(Hingorani et al., 2005; Morton et al., 2010). Mixed male and female populations were used for each genotype. Cohorts were on a mixed strain background but all cohorts consisted of litter-matched controls and were killed at a humane clinical end point. For allograft of mixed background KPC cells, CD-1 Nude (Crl:CD1-Foxn1^{nu}) female mice were used (Charles River, 7 weeks old). For allograft of pure C57Bl6/J KPC cells, C57Bl6/J female mice were used (Charles River, 7 weeks old). For tumor growth experiment (Figures 4E and 4F) BALB/c Nude female mice (CAnN.Cg-Foxn1^{nu}/Crl) were used (Charles River, 8 weeks old).

METHOD DETAILS

Extraction of ImPSC cell lines

ImPSC #1 cells were a gift from Jurre Kamphorst(Auciello et al., 2019). Briefly, healthy pancreas tissue extracted from C57Bl6/J mice was minced and digested for 20mins at 37°C with 0.1% DNase (Roche), 0.05% Collagenase P (Roche) and 0.02% Pronase (Roche) in Gey's balanced salt solution (GBSS; Sigma Aldrich). The tissue was then titurated until the large pieces were no longer visible, passed through a 100μm filter and washed with GBSS. The cells were then pelleted and resuspended in 9.5ml GBSS with 0.3% BSA and 8ml Nycodenz solution (Sigma Aldrich). The cell suspension was layered beneath GBSS containing 0.3% BSA, and centrifuged at 1400 x g for 20 min at 4°C. Stellate cells were harvested from the interface of the Nycodenz solution at the bottom and the aqueous solution at the top. The PSCs isolated were then washed with GBSS and resuspended in DMEM with 10% characterized FBS (HyClone), 100 U/ml penicillin and 100μ/ml streptomycin (Invitrogen). The cells were immortalized with the pRetro. Super.shARF retroviral plasmid (provided by the Karen Vousden lab) and selected with blasticidin (4μM).

ImPSC #2 & #3 lines were isolated using a very similar protocol as ImPSC #1 with some minor differences detailed below. Pancreas tissue was extracted from Pdgfra$^{tm11(EGFP)Sor}$ mice(Hamilton et al., 2003) (JAX stock #007669), minced with a scalpel and digested with 0.1% DNase I (Thermo Fisher Scientific, 90083) and 0.05% collagenase P (Roche, 11 213 865 001) in GBSS for 30mins at 37°C. The solution was then passed through a 100μm filter, washed with GBSS, pelleted and resuspended in 6ml GBSS containing 0.3% BSA. The cell suspension was then mixed with 8ml Histodenz solution (43.75% in GBSS), layered beneath GBSS containing 0.3% BSA, and centrifuged at 1400 x g for 20mins at 4°C. Stellate cells were harvested from the interface of the Histodenz solution at the

bottom and the aqueous solution at the top. The PSCs were washed in PBS containing 3% FBS and resuspended in DMEM containing 10% FBS, 1% penicillin- streptomycin, 0.2% amphotericin B and glutamine (2mM). After culture was established, fibroblasts expressing GFP were isolated via FACS and immortalized spontaneously.

ImPSC #1 cells stably expressing GFP (ImPSC-GFP cells) were generated by the PiggyBac transposon system. Briefly, 5×10^4 ImPSC #1 cells were seeded in a 6-well plate. 24h after seeding, cells were transfected using Lipofectamine 3000 (Thermo Fisher Scientific, LS3000008) with 1.5μg Super piggyBac Transposase expression vector (Cambridge Biosciences, PB210PA-1) and 0.6μg PB-GFP PB-CMV-MCS-EF1-GreenPuro (Cambridge Biosciences, PB513B-1). 24h after transfection, cells were selected in 5μg/ml puromycin for 48h, until puromycin sensitive control cells treated in parallel were dead.

Production of KPC-EV and KPC-IDO1 cell lines

Pure C57Bl6/J KPC cells stably expressing IDO1-RFP or RFP only (empty vector control) were generated using the piggyback system. Briefly, human IDO1 cDNA was cloned into the PB-RFP PB-CMV-MCS-EF1-RedPuro cDNA cloning and expression vector using XbaI and EcoRI. Successful cloning was confirmed by full sequencing of the insert. 2.5×10^5 pure C57Bl6/J KPC cells were seeded in a 6-well plate. 24h after seeding cells were transfected using Lipofectamine 3000 (Thermo Fisher Scientific, LS3000008) with 1.5μg Super piggyBac Transposase expression vector (Cambridge Biosciences, PB210PA-1) and 0.6μg of either PB-RFP PB-CMV-IDO1-EF1-RedPuro (IDO1 stable expression) or PB-RFP PB-CMV-MCS-EF1-RedPuro (empty vector control, Cambridge Biosciences, PB514B-2). 24h after transfection, cells were selected in 5μg/ml puromycin for 48h, until puromycin sensitive control cells treated in parallel were dead. To identify high expressers of IDO1, cells were grown as clones and validated for expression by immunoblotting.

Hypoxia experiments

Cells were seeded and allowed to grow for 48h to ~80% confluence under normal tissue culture conditions. Cells were then transferred to a humidified Whitley H35 hypoxystation (Don Whitley Scientific) controlled by a hypoxic gas mixer at 37°C with 1% O_2, 5% CO_2, and 94% N_2 for 24h prior to lysis following standard RIPA lysis protocol (see below)

Attachment independent 3D growth experiments

1×10^6 cells were seeded in ultra-low attachment plates (Corning Costar, CLS3471) for 48h. For treatments, cells were collected, centrifuged at 50 x g for 5mins and washed in PBS. Cells were then resuspended in 2ml of treatment medium and transferred back into ultra-low attachment plates for indicated treatment times.

Conditioned medium experiments

9×10^6 HPAF-II or CFPAC-1 cells were seeded in 10cm dishes in their normal growth media. Experimental media for conditioning were formulated lacking tryptophan and supplemented with the stated concentrations of $^{13}C_{11}$-tryptophan. After 48h in culture, cells were washed in PBS and media for conditioning was added. After 48h, conditioned medium was collected and passed through a 0.45μm filter to remove cells. Conditioned medium was stored at −20°C prior to use.

Co-culture experiments

5×10^5 ImPSC-GFP cells were seeded either alone or with 1×10^4 CFPAC-1 cells in 6-well plates. Experimental media was formulated lacking tryptophan and supplemented with 0.4mM $^{13}C_{11}$ tryptophan. After 24h in culture, cells were washed in PBS and media containing human IFNγ (1ng/ml) or vehicle only control and/or epacadostat (1μM) or vehicle only control was added. After 24h, cells were detached rypsinizationion, washed in PBS and resuspended to a concentration of 1×10^7 cells/ml in cold supplemented PBS (PBS + 3% FBS, 5mM glucose, MEM Amino Acids (Life Technologies, 11130-036) & MEM NEAA (Life technologies, 11140-035)). The cell suspensions were then passed through a 70μm mesh (Fisher Scientific, PRN-020-030V) to ensure a single-cell suspension and subjected to fluorescence-activated cell sorting (FACS) using an Aria sorter Z6001 (BD Biosciences) to separate GFP-positive cells from unlabelled cells. The resultant cell suspension was centrifuged at 300 x g for 5mins and the pellet was resuspended in ice-cold lysis solvent. Using the cell counts obtained from FACS, the volume of lysis solvent was normalized to 2×10^6 cells per ml. Subsequent isolation of metabolites for LCMS was performed as below.

RNA extraction, reverse transcription, and expression analysis

The extraction and purification of total RNA was performed using the RNAeasy purification Kit (QIAGEN) combined with RNase-Free DNase (QIAGEN) treatment according to the manufacturer's protocol. RNA concentration and quality was determined using the NanoDrop 2000 spectrophotometer before downstream processing. Reverse transcription of isolated RNA was performed using the High Capacity cDNA Kit (Applied Biosystem) according to the manufacturer's instructions. For expression analysis cDNA was diluted in DNase- and RNase-free water. qRT-PCR was performed on a CFX96 Touch Real-Time PCR Detection System in 96-well plates. cDNA was added to Brilliant III Ultra-Fast SYBR® Green QPCR Master Mix (Agilent) and primers (1 μM final concentration) in a total reaction volume of 10 μL. qRT-PCR thermal program was carried out by an initial denaturation/polymerase activation step at 95°C for 10 minutes followed by 40 cycles of amplification (95°C for 15 s and 60°C for 45 s) and melting curve. Fluorescent signal was detected at the end of every amplification step and continuously during the melting curve. Amplification

curves were analyzed using the Biorad CFX Manager software and the $2^{-\Delta\Delta CT}$ method was used to normalize expression to GAPDH and ACTN. The following primers we used: IDO1 (Fw: 5′- CTCAGCATTGATCATCTCAC; Rev: 5′- AAGACACAGTCTGCATAAAC), ACTIN (Fw: 5′- CCAACCGCGAGAAGATGA; Rev: 5′- CCAGAGGCGTACAGGGATAG), GAPDH (Fw: 5′- AGCCACATCGCTCAGAC AC; Rev: 5′- GCCCAATACGACCAAATC).

RNA-Scope *in situ* hybridization and Immunohistochemistry

Ido1 RNA expression across tissue sections was quantified by *in situ* hybridization (ISH) as described below. In order to define which regions of the tissue sections were cancer cells and which were stromal cells we stained contiguous sections for the tumor cell marker EpCAM (Anti-EpCAM antibody, Abcam ab71916) by immunohistochemistry (IHC).

Tumor tissue samples were fixed in 10% neutral buffered formalin then embedded in paraffin to produce a formalin fixed paraffin embedded (FFPE) block. Prior to staining 4μm sections were heated in an oven at 60°C for 2 hours. Sections for EpCAM IHC staining were loaded into an Agilent PT module and dewaxing/epitope retrieval took place at 97°C for 20 minutes using Target Retrieval Solution, high pH (Agilent, Denmark). After epitope retrieval all sections were rinsed in flex wash buffer (Agilent, Denmark) prior to being loaded onto the autostainer. The sections then underwent peroxidase blocking (Agilent, Denmark) for 5 minutes, washed with flex buffer before application of primary antibody at a previously optimized dilution (1/1500) for 40 minutes. The sections were then washed with flex wash buffer before application of appropriate secondary antibody for 30 minutes (Rabbit EnVision, Agilent, UK). Sections were rinsed with flex wash buffer applying Liquid DAB (Agilent, UK) for 10 minutes. The sections were then washed in water, counterstained with hematoxylin z and coverslipped using DPX mountant (CellPath, UK).

ISH detection for Ido1 (product code 315978) and PPIB (product code 313918) both from Advanced Cell Diagnostics (Hayward, CA) mRNA was performed using RNA-Scope 2.5 LS (Brown) detection kit (Advanced Cell Diagnostics, Hayward, CA). This technique was performed on a Leica Bond Rx autostainer strictly adhering to the manufacturer's instructions. Slides were digitally imaged using a Leica Aperio AT2 (Leica Biosystems), full cross section digital images were uploaded to HALO Software v3 (Indica Labs). Regions of cancer cells and stroma were defined based on EpCAM staining and Ido1 RNA ISH signals were quantified in these regions across the full cross section.

Survival analysis

Clinical and normalized gene expression (FPKM) data of TCGA-PAAD dataset were obtained from the Human Protein Atlas (https://www.proteinatlas.org/). The expression cut-off was calculated on the Human Protein Atlas web portal and used for Kaplan-Meier curve plotting using GraphPad Prism Software v9. Survival analysis was performed by using the Mantel-Cox log-rank test.

Western blotting

Protein was extracted from whole cells by lysis in RIPA buffer (Thermo Fisher Scientific, 89900) supplemented with protease and phosphate inhibitor cocktail (Thermo Fisher Scientific, A32959). For cells grown in ultra-low attachment plates, cells were collected by centrifugation at 50 x g for 5mins, washed in PBS and resuspended in 100μL RIPA lysis buffer. Cells were left to lyse on ice for 10mins and then homogenized by pipetting. For adherent cells, cells were washed in PBS and lysed in 200μL RIPA lysis buffer on ice *in situ*, collected using a cell scraper and homogenized by pipetting. Tissue samples were snap-frozen and stored at −80°C. Frozen samples were weighed before lysis to ensure a minimum sample size of 20mg. Samples were homogenized in 2ml RIPA lysis buffer using a TissueLyser II (QIAGEN). Lysates were cleared by centrifugation at 12,000 x g for 15mins at 4°C. Supernatants were collected and total protein content quantified by BCA assay (Thermo Fisher Scientific, 23227). Lysates were normalized by total protein content and prepared for western blotting with the addition of 4X Bolt LDS Sample Buffer (+ 355mM β-mercaptoethanol) and heated to 95°C for 10mins. Lysates (25μg) were resolved on Bolt 4%–12% bis-tris plus pre-cast gels (Thermo Fisher Scientific, NW04122BOX & NW04125BOX) using Bolt MOPS SDS Running Buffer running buffer (Thermo Fisher Scientific, B0001) and transferred to nitrocellulose membranes. When total protein staining was performed, it was done prior to blocking using Revert Total Protein Stain (LI-COR, 926-11010). Membranes were blocked for 1 hour using Odyssey® Blocking Buffer (TBS) (LI-COR, 927-50000) and incubated overnight at 4°C with primary antibodies. All primary antibodies were diluted in Odyssey® Blocking Buffer at a concentration of 1:1000, except actin, which was used at 1:10,000. Membranes were washed three times in TBS + 1% TWEEN® 20 and incubated with secondary antibodies (1:10,000) for 1h at room temperature. Fluorescence intensity was captured and quantified using a LI-COR Odyssey® Fc Imaging System with Image Studio software (version 5.2). See Key resources table for list of antibodies.

In vivo models

LSL-Kras$^{G12D/+}$, Pdx1-cre;LSL-Kras$^{G12D/+}$;Trp53$^{fl/+}$ and Pdx1-cre;LSL-Kras$^{G12D/+}$;LSL-Trp53$^{R172H/+}$ mice were allowed to develop tumors, killed at humane clinical endpoint and tumors removed for analysis. Pancreas tissue from healthy non-cre-expressing littermates were used as controls.

For allograft experiments assessing Ido1 expression *in vivo* (Figure 1): three separate C57Bl6/J KPC primary cell lines were implanted by bilateral subcutaneous injections (2×10^6 cells per injection) into female C57Bl6/J or female Crl:CD1-Foxn1^{nu} (CD1-Nude) mice. Mixed background KPC cells were implanted by unilateral subcutaneous injections (2×10^6 cells per flank) into Crl:CD1-Foxn1^{nu} (CD1-Nude) female mice. Mice were monitored daily until they reached clinical end point or tumor size reached 300mm^3.

For allograft experiments assessing $^{13}C_{11}$-tryptophan labeling *in vivo* (Figures 4B and S4A–S4C): KPC-IDO1 and KPC-EV cells (described above) were implanted by unilateral subcutaneous injections (2 × 10^6 cells per injection) into female pure C57Bl6/J mice and allowed to form tumors of 300-500mm^2. For $^{13}C_{11}$-tryptophan tracing mice were fasted for 3h and then received an intraperitoneal injection of 800μl of 120mM ^{13}C Tryptophan (Cambridge Isotope Laboratories). 3h after injection, mice were killed and tumors removed for LCMS analysis.

For allograft experiment assessing tumor growth (Figures 4E and 4F): KPC-IDO1 cells were implanted by unilateral subcutaneous injections (1 × 10^6 cells per injection) into female BALB/c nude mice. One day after cell were implanted mice were transferred to a control diet (containing serine and glycine) or a matched diet lacking serine and glycine as described previously(Maddocks et al., 2017). After 4 days on diet mice received vehicle only (0.5% hydroxypropylmethylcellulose, 0.25% tween-20 (both Sigma) in water, or 50mg/kg of epacadostat by oral gavage twice a day. Tumors were measured three times per week by caliper, and volumes were calculated using the formula: volume = (length x width^2) / 2. On reaching the maximum permitted tumor size (tumor length or width ≥ 15mm) or if tumors became ulcerated, mice were humanely killed.

LCMS for steady-state metabolite measurements

Metabolomics experiments were performed as described previously(Maddocks et al., 2017). Cells were seeded into 6-well plates in complete medium and allowed to grow to ~80% confluence. Cells were washed with PBS and the relevant experimental media were added for the stated times. Duplicate wells were used for cell counting: cell counts (2D cells) or protein concentration (3D cells BCA assay) were used to normalize the volume of lysis solvent prior to metabolite extractions (1 × 10^6 cells per ml). For 2D grown cells, cells were washed quickly in PBS, then ice-cold lysis solvent (Methanol 50%, acetonitrile 30%, water 20%) was added and cells scraped on ice. For 3D grown cells, cells were transferred to 15ml falcon tubes and centrifuged at 50 x g for 5 minutes. The supernatant was removed and the cell pellet was washed in PBS and centrifuged again. The supernatant was removed and the cell pellet resuspended in ice-cold lysis solvent. Lysates were transferred to 1.5ml tubes on ice, vortexed, then centrifuged at 18,000 x g at 4°C for 10mins. Supernatants were collected and stored at −80°C for LCMS analysis. Tissue samples were snap-frozen and stored at −80°C. Frozen samples were weighed before lysis. Samples were homogenized in 2ml ice cold lysis solvent using a TissueLyser II (QIAGEN). Lysates were then cleared of protein by centrifugation at 18,000 x g for 10mins at 4°C and then normalized to 10mg/mL with lysis buffer based on original tissue mass.

Sample analysis was performed using an LCMS platform consisting of an Accela 600 LC system and an Exactive mass spectrometer (Thermo Scientific). A Sequant ZIC-pHILIC column (4.6mm x 150mm, 3.5 μm) (Merck) was used to separate the metabolites with the mobile phase mixed by A = 0.1% (v/v) formic acid in water and B = 0.1% (v/v) formic acid in acetonitrile. A gradient program starting at 20% of A and linearly increasing to 80% at 30 min was used followed by washing (92% of A for 5 mins) and re-equilibration (20% of A for 10min) steps. The total run time of the method was 45 min. The LC stream was desolvated and ionised in the HESI probe. The Exactive mass spectrometer was operated in full scan mode over a mass range of 70–1,200 m/z at a resolution of 50,000 with polarity switching. The LCMS raw data was converted into mzML files by using ProteoWizard and imported to MZMine 2.10 for peak extraction and sample alignment. A house-made database including all possible ^{13}C and ^{15}N isotopic m/z values of the relevant metabolites was used for the assignment of LCMS signals. Finally the peak areas were used for comparative quantification.

GCMS for formate analysis

Gas chromatography-mass spectrometry (GCMS) (Agilent) was used to quantify formate, as described previously(Meiser et al., 2016). 40μL of sample was added to 20μL of the internal standard d_2-formate (50 μM), 50μL pyridine, 10μl NaOH (1N) and 5μL benzyl alcohol. While vortexing, 20 μL of methyl choroformate was added to this mixture for derivatisation. 100μL methyl tertiary butyl ether (MTBE) and 200μL H_2O were then added, and the samples subsequently vortexed for 10 s and centrifuged for 10mins at maximum speed. The apolar phase was then transferred to a GC-vial and capped. Standards and blank samples (water) were prepared in the same manner and analyzed with the experimental samples to subtract the background and validate the quantification. MassHunter Quantitative analysis software (version B.06.00 – Agilent Technologies) was used to extract and process the peak areas for formate, d_2-formate and ^{13}C formate. After correction for background signals, quantification was performed by comparing the peak area of formate (m/z of 136) and of ^{13}C formate (m/z of 137) against that of d_2-formate (m/z of 138).

Carbon-13 (^{13}C) labeling of metabolites

Experimental media were formulated lacking tryptophan or serine and supplemented with the stated concentrations of $^{13}C_{11}$-tryptophan (Cambridge Isotope Laboratories Inc., CLM-4290-H-PK), $^{13}C_3{}^{15}N_1$-serine (Cambridge Isotope Laboratories Inc., CNLM-474-H-PK) or $^{13}C_1$-formate (Cambridge Isotope Laboratories Inc., CLM-1284-PK). The same basic protocol was used as for steady state metabolite measurements (see above). Cell culture, harvest, metabolite extraction and analysis by LCMS was performed as described above. Labeled isotopologues were defined as mass + *n* (m+*n*), where n = number of ^{13}C incorporated. The unlabelled form is defined as m+0 (i.e., zero ^{13}C present). To correct data for natural (background) ^{13}C abundance we used unlabelled controls (i.e., complete medium without ^{13}C tracer present) and used the ^{13}C peak areas detected in these samples as empirical ^{13}C baselines which were subtracted from experimental samples.

E.g. Calculation of baseline ^{13}C fraction for serine using ^{12}C control sample: (PA = peak area)

CellPress
OPEN ACCESS

$$m+1^{PA}/(m+0^{PA}+m+1^{PA}) = \text{Baseline}^{13}\text{C fraction}$$

E.g. Calculation of ^{13}C fraction for serine labeling in an experimental (^{13}C labeled) sample:

$$(m+1^{PA}/\ (m+0^{PA}+m+1^{PA})\ ) - \text{Baseline}^{13}\text{C fraction} = {}^{13}\text{C fraction}\ (m+1)$$

For simplicity we have presented the sum of the ^{13}C labeled fraction for m+*n* peaks detected above baseline and define these in the x axis of each bar chart. Matched plots where each isotopologue is plotted separately are presented in supplemental data.

QUANTIFICATION AND STATISTICAL ANALYSIS

Statistical analysis for Figures 4E and 4F was calculated using Microsoft Excel for Mac (v16.43), all other statistical analyses were calculated using GraphPad Prism Software v9. For *in vivo* experiments the numbers of mice are shown in each Figure / Figure Legend. For statistical comparisons of groups of mice (e.g., for tumor protein expression [Figure 1C] or tumor growth [Figures 4E and 4F]) unpaired 2-sided t tests were performed. For statistical analysis of protein expression by western blot (Figure 1C) single blots were performed on multiple independent experiments (n = 3-4), as defined in Figures / Figure Legends. For statistical comparisons of protein expression in 2D and 3D growth conditions *in vitro*, single blots from n = 3-4 independent experiments were quantified and compared using paired 2-sided t tests (Figures 2F and S2B). For comparison of RNA-Scope (cancer versus stromal cell IDO1 expression, [Figure S5C]) a paired 2-sided t test was used. For *in vitro* mass spectrometry experiments n = 3 biological replicates – corresponding to three separate wells of cells – were used throughout. Error bars are Standard Deviation (SD), a descriptive measure of variability.

Molecular Cell, Volume *81*

Supplemental information

Immune-regulated IDO1-dependent tryptophan metabolism is source of one-carbon units for pancreatic cancer and stellate cells

Alice Clare Newman, Mattia Falcone, Alejandro Huerta Uribe, Tong Zhang, Dimitris Athineos, Matthias Pietzke, Alexei Vazquez, Karen Blyth, and Oliver David Kenneth Maddocks

Supplemental Figure 1.

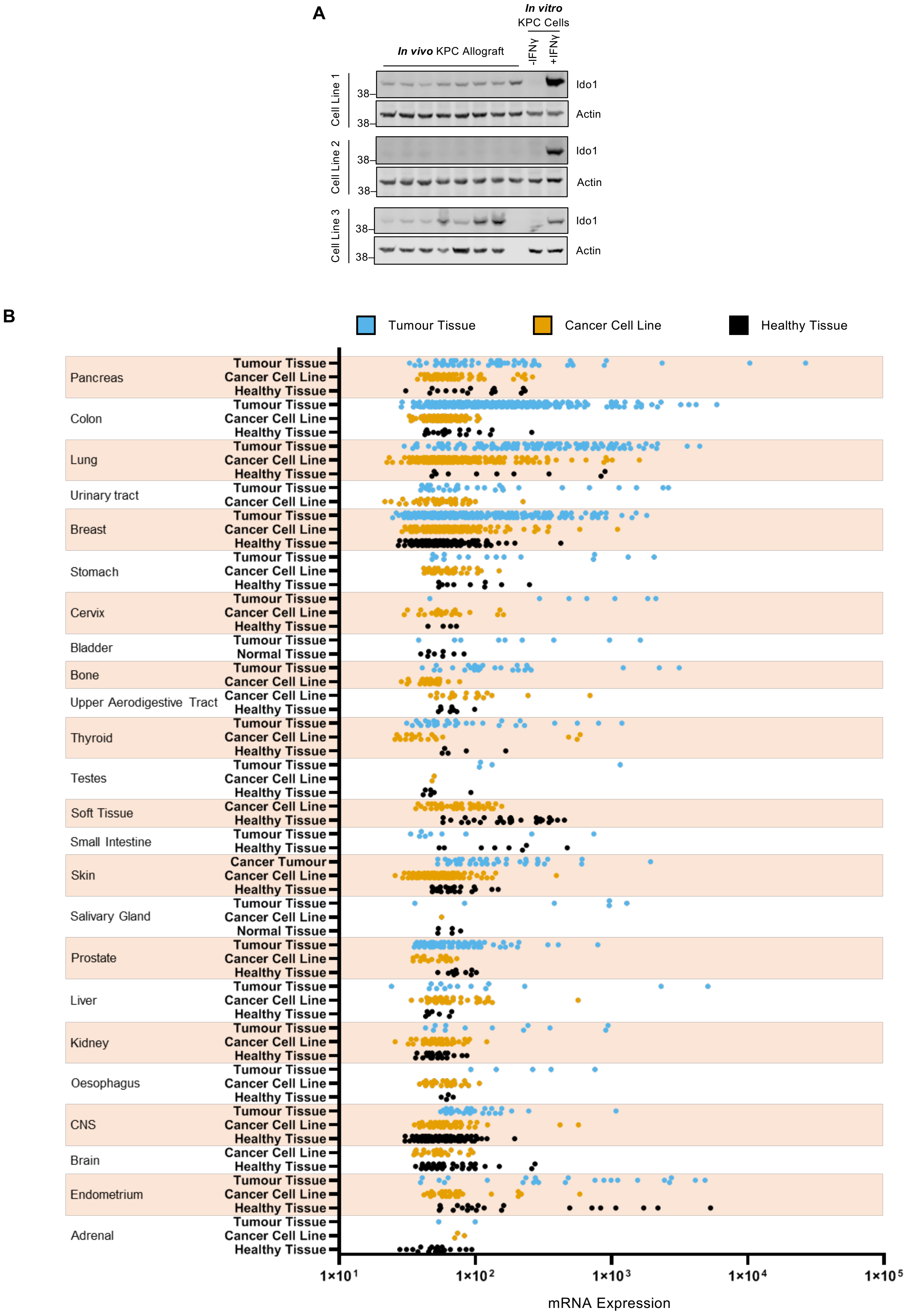

Supplemental Figure 1.

IDO1 expression in mouse and human tumours. Related to Figure 1.

A, KPC cells were isolated from C57Bl6/J *Pdx1*-cre;*Kras*$^{G12D/+}$;*Trp53*$^{R172H/+}$ mice and either treated in culture with mouse IFNγ (1ng/ml) for 24h or subcutaneously injected into the flank of C57Bl6/J mice to form tumours. Cell and tumour lysates were subjected to western blot for the indicated proteins. **B**, Data extracted from the MERAV database showing the relative abundance of IDO1 mRNA from microarray data across a range of human cell lines and tumour tissue (http://merav.wi.mit.edu/; Shaul, Y.D., Yuan, B., Thiru, P., Nutter-Upham, A., McCallum, S., Lanzkron, C., Bell, G.W., and Sabatini, D.M. (2016). MERAV: a tool for comparing gene expression across human tissues and cell types. Nucleic acids research *44*, D560-566).

Supplemental Figure 2.

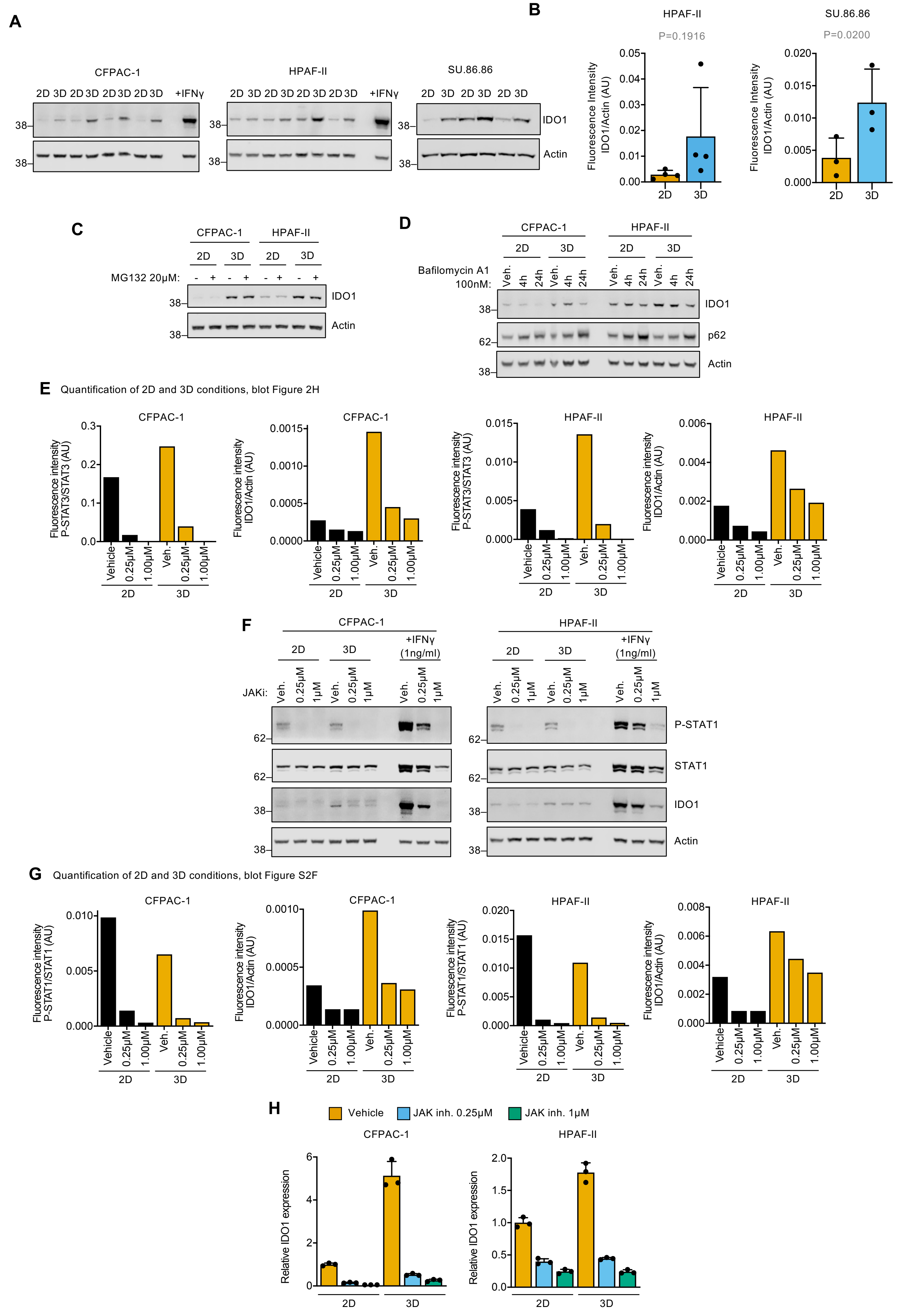

Supplemental Figure 2.

Regulation of IDO1 expression by attachment independent 3D growth. Related to Figure 2.

A, B, CFPAC-1, HPAF-II and SU.86.86 cells were either growth in normal tissue culture plates (2D) or in ultra-low-attachment tissue culture plates (3D) for 24h, or cultured in 2D and treated with 1ng/ml IFNγ. Lysates were (**A**) analysed by western blot for the indicated proteins and (**B**) band intensity of IDO1 relative to actin (loading control) quantified using LiCor infra-red scanner (n=3 or 4 independent experiments, paired 2-sided t-test, P-value shown, bars = SD). **C-E**, CFPAC-1 and HPAF-II cells were grown in 2D or 3D conditions for 24 hours and lysates analysed by western blot for indicated proteins (**C**) after 16h treatment with proteasome inhibitor MG132 (20μM) or vehicle-only control (**D**) after treatment for the indicated times with lysosome inhibitor bafilomycin A1 (100nM) or vehicle-only control (p62 used as positive control for lysosomal regulation). **E,** Quantification of IDO1 and phospho-STAT3 band intensities using LiCor infra-red laser scanner for blot shown in Figure 2H. **F,** CFPAC-1 and HPAF-II cells were grown in 2D or 3D conditions for 24 hours and lysates analysed by western blot for indicated proteins after 16h treatment with JAKi (at indicated concentrations), vehicle-only control or IFNγ (1ng/ml). **G**, Quantification of IDO1 and phospho-STAT1 band intensities using LiCor infra-red laser scanner for blot shown in Figure S2F. **H**, mRNA was extracted from CFPAC-1 and HPAF-II cells and subjected to qRT-PCR (n=3 biological replicates, bars = SD).

Supplemental Figure 3.

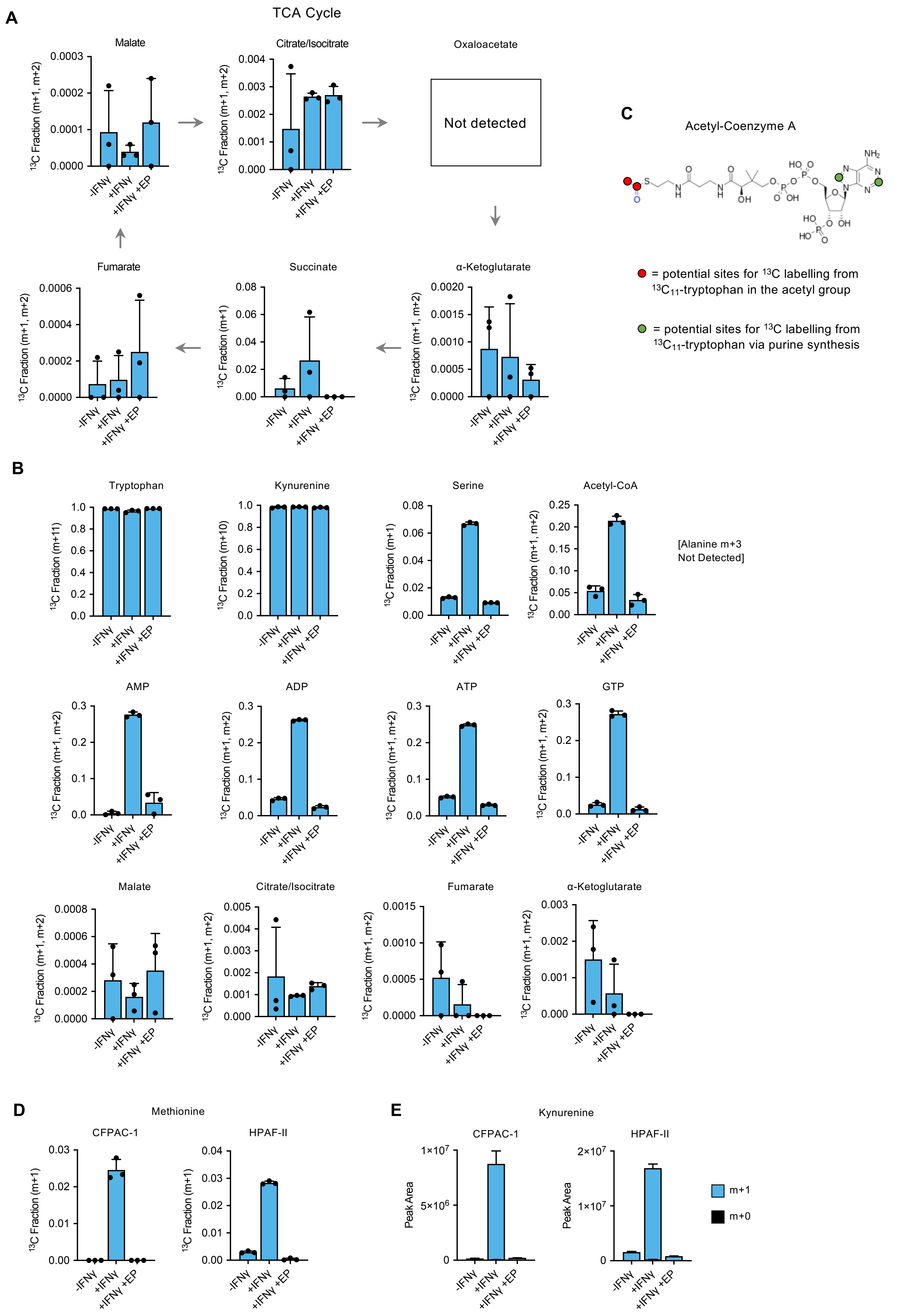

Supplemental Figure 3.

IDO1-dependent tryptophan metabolism analysed by LCMS. Related to Figure 3.

A & B, CFPAC-1 (**A**) and HPAF-II (**B**) cells were grown with or without human IFNγ (1ng/ml), or with IFNγ and IDO1 inhibitor Epacadostat for 24h in in the presence of $^{13}C_{11}$-tryptophan. Intracellular metabolites were analysed by LCMS and corrected for natural ^{13}C abundance (n=3 biological replicates, bars = SD). **C**, The potential positions of tryptophan derived carbons are shown in a structural diagram of Acetyl-CoA. **D,** methionine levels detected by LCMS in the same experiments described in A & B above. **E**, Raw peak area values for kynurenine detected by LCMS are shown for the same experiments described in A & B above.

Supplemental Figure 4.

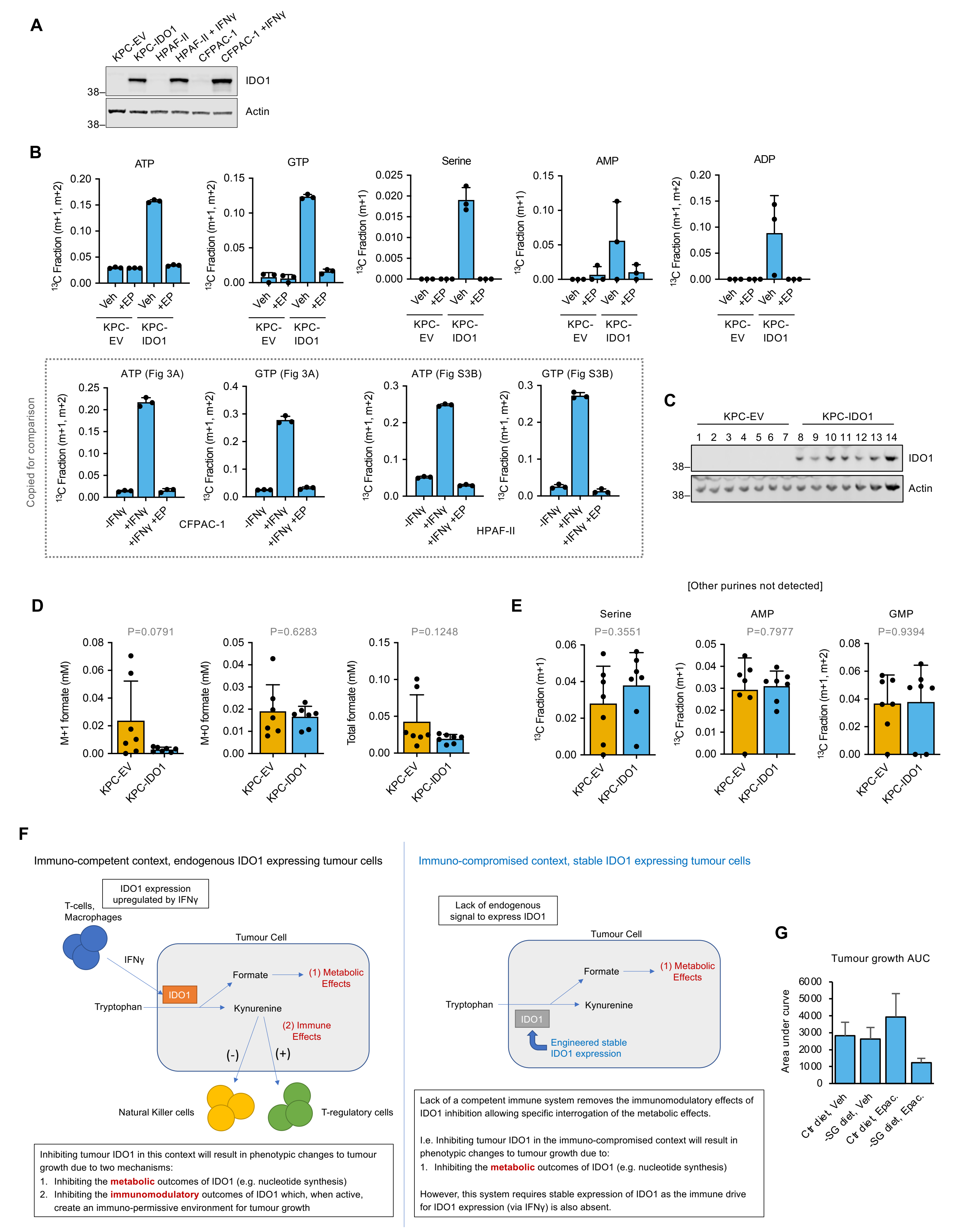

Supplemental Figure 4.

***In vivo* tryptophan metabolism. Related to Figure 4.**

A, KPC cell line 2 (Figure 1E & S1A) from C57Bl6/J Pdx1-cre;*Kras*$^{G12D/+}$;*Trp53*$^{R172H/+}$ mice were engineered to stably express IDO1 (KPC-IDO1) or empty-vector control (KPC-EV), protein lysates from cells were analysed by western blot for IDO1 expression alongside CFPAC-1 and HPAF-II cells which had been treated with human IFNγ (1ng/ml) for 24h. **B**, KPC-EV and KPC-IDO1 cells were grown with or without IDO1 inhibitor Epacadostat for 24h in in the presence of $^{13}C_{11}$-tryptophan. Intracellular metabolites were analysed by LCMS and corrected for natural ^{13}C abundance (n=3 biological replicates, bars = SD). ATP and GTP LCMS data for IFNγ/epacadostat treated CFPAC-1 and HPAF-II cells (copied from Figures 3A and S3B) is shown for comparison. **C-E**, KPC-EV and KPC-IDO1 cells were injected subcutaneously into the flanks of C57Bl6/J mice (as shown and used in Figure 4A &B), once tumours had formed the mice were given a single by intraperitoneal injection of 800µl 120mM $^{13}C_{11}$ tryptophan and harvested after 3h. Tumour tissue was excised and analysed by immunoblotting for the indicated proteins (**C**). Serum was analysed by GCMS for formate (**D**) and LCMS for serine and purine nucleotides (**E**) (n=7 mice in each group, bars are SD). **F,** Cartoon illustrating the rationale for use of immunocompromised mice with stable IDO1-expressing (KPC-IDO1) tumour cells to specifically interrogate the metabolic effects of IDO1 inhibition on tumour growth. **G,** Area under the curve analysis was performed on the tumour volume data presented in Figure 4E & F, bars are standard error of mean.

Supplemental Figure 5.

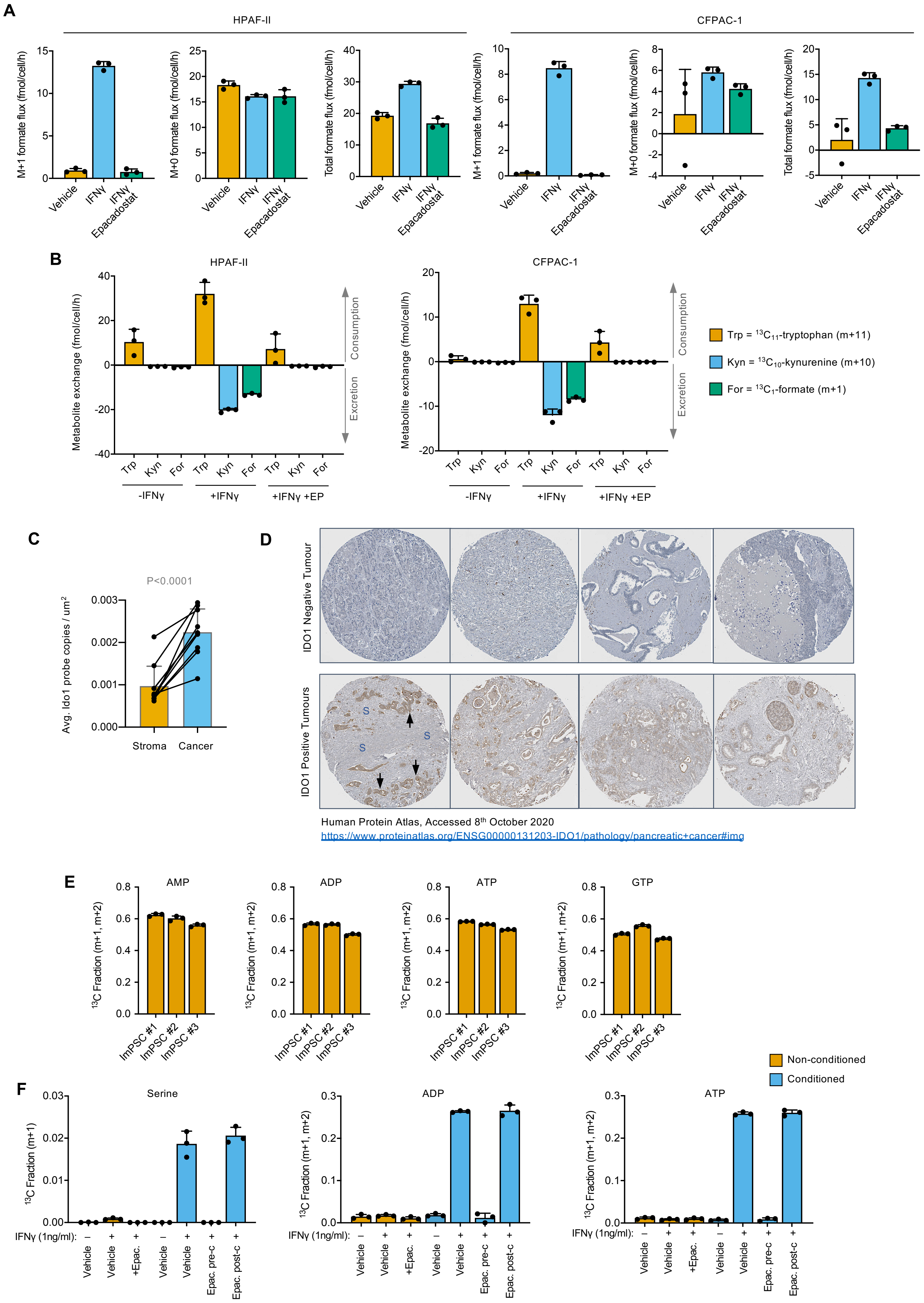

Supplemental Figure 5.

Formate metabolism in PDAC and pancreatic stellate cells. Related to Figure 5.

A & B, HPAF-II and CFPAC-1 cells were treated with IFNγ (1ng/ml) with or without epacadostat (1μM) and/or vehicle only controls in the presence of $^{13}C_{11}$-tryptophan for 24h. Cell were grown in complete medium containing serine and glycine (both 0.4mM) and $^{13}C_{11}$-tryptophan 0.4mM. Media samples were analysed by a targeted GCMS for extracellular formate (A) and by LCMS for tryptophan and kynurenine (B). (n=3 biological replicates, bars = SD). GCMS data for unlabelled (m+0), labelled (m+1) and total (m+0 + m+1) formate is shown in (A). LCMS data for labelled tryptophan (m+11) and kynurenine (m+10) are shown in (B), alongside labelled formate (m+1) data reproduced from (A) for comparison. **C,** Formalin fixed paraffin embedded tissue sections from tumours shown in Figure 1B were analysed by RNA-Scope for IDO1 transcript expression (n=10 tumours, paired 2-sided T-test, P-value shown). Contiguous tissue sections were stained by immunohistochemistry for a cancer cell marker (EpCAM) to differentiate stromal cells from cancer cells. **D,** Images of pancreatic cancer tumours stained by immunohistochemistry for IDO1 were downloaded from the Human Protein Atlas. 'S' denotes stroma, arrow heads indicate PDAC cells embedded within the stroma **E**, Three separate immortalised murine pancreatic stellate cell lines (ImPSC#1, ImPSC#2 & ImPSC#3) were cultured for 24h in the presence of $^{13}C_1$-formate and intracellular metabolites were analysed by LCMS (n=3 biological replicates, bars = SD). **F**, CFPAC-1 cells were treated with IFNγ (1ng/ml) and/or epacadostat (1μM) and/or vehicle only in the presence of $^{13}C_{11}$-tryptophan. Conditioned media were collected after 24h and ImPSC#2 cells were cultured in this media, or in non-conditioned treatment-matched media. After 24h, intracellular metabolites were analysed by LCMS (n=3 biological replicates, bars = SD).
